# Unraveling Genomic Regions Controlling Root Traits as a Function of Nitrogen Availability in the MAGIC Wheat Population WM-800

**DOI:** 10.3390/plants11243520

**Published:** 2022-12-14

**Authors:** Laura Schmidt, Kerstin A. Nagel, Anna Galinski, Wiebke Sannemann, Klaus Pillen, Andreas Maurer

**Affiliations:** 1 Chair of Plant Breeding, Institute of Agricultural and Nutritional Sciences, Martin Luther University Halle-Wittenberg, Betty-Heimann-Str. 3, 06120 Halle, Germany; 2 IBG-2: Plant Sciences, Institute of Bio- and Geosciences, Research Institute Jülich GmbH, 52425 Jülich, Germany

**Keywords:** nitrogen uptake efficiency (NUpE), root system architecture (RSA), GWAS, QTL, winter wheat, haplotype, multiparental population (MAGIC), high-throughput phenotyping

## Abstract

An ever-growing world population demands to be fed in the future and environmental protection and climate change need to be taken into account. An important factor here is nitrogen uptake efficiency (NUpE), which is influenced by the root system (the interface between plant and soil). To understand the natural variation of root system architecture (RSA) as a function of nitrogen (N) availability, a subset of the multiparent advanced generation intercross (MAGIC) winter wheat population WM-800 was phenotyped under two contrasting N treatments in a high-throughput phenotyping system at the seedling stage. Fourteen root and shoot traits were measured. Subsequently, these traits were genetically analyzed using 13,060 polymorphic haplotypes and SNPs in a genome-wide association study (GWAS). In total, 64 quantitative trait loci (QTL) were detected; 60 of them were N treatment specific. Candidate genes for the detected QTL included *NRT1.1* and genes involved in stress signaling under N−, whereas candidate genes under N+ were more associated with general growth, such as *mei2* and *TaWOX11b*. This finding may indicate (i) a disparity of the genetic control of root development under low and high N supply and, furthermore, (ii) the need for an N specific selection of genes and genotypes in breeding new wheat cultivars with improved NUpE.

## 1. Introduction

Wheat (*Triticum aestivum*) is one of the most important crops in human nutrition [1] but currently also requires a high level of nitrogen (N) fertilization, because N plays a central role for plant growth and the formation of yield and quality in winter wheat [2]. On the downside, intensive N fertilization is known to have negative effects on biodiversity [3,4,5], and leads both to the degradation of land and water [6,7] (causing eutrophication and hypoxic zones in marine ecosystems) and to the emission of greenhouse gases, which contributes to ozone damage and global warming [8,9].

At this point, nutrient use efficiency (NUE) enters the equation, being defined as grain dry matter yield per unit N available in the soil, consisting of N uptake efficiency (plant N uptake/available N; NUpE) and N utilization efficiency (grain dry matter yield/N plant uptake; UTE) [10].

Efficient N uptake could prevent these negative effects of intensive N fertilization, and a well-developed root system (already at the seedling stage) plays a crucial role in the NUE of plants [2,11,12,13], acting as an interface between the plant and water and nutrients in the soil [13,14]. Fortunately, winter wheat has one of the fastest growing and prolific root systems of all arable crops [15]. The size of a root system as well as its architecture is known to exhibit a very high phenotypic plasticity in response to environmental factors [16,17,18,19], particularly with respect to nitrogen availability and distribution [20]. However, in addition to environmental effects, there is also genotypic variation in the development of the root system architecture (RSA) of wheat [21], providing the baseline for later adaptation to the environmental conditions. To investigate seedling roots, soil-free methods, such as hydroponic or paper-based approaches, are often preferred [22,23,24], because root characteristics can be examined more conveniently. However, a drawback of these soil-free methods is the lack of the complex environmental interaction effects just mentioned, which can only be represented to a limited extent in such setups.

Studies have made it evident that a well-developed root system can, in general, improve N uptake, but it must be taken into consideration that the formation and maintenance of the root system is a carbon-costly process [14,25]. Under N deficiency, wheat invests particularly in root length in order to acquire N resources [26]. Especially the growth of lateral roots is of importance here [27], which is regulated by N signaling via a NO_3_-regulated repression [13,28]. In this regard, *NRT1.1*, a dual-affinity nitrate transporter, plays a key role not only in the transport of N but also in the signaling, which leads to N dependent changes in downstream gene expression. *NRT1.1,* in its function as a nitrate sensor, was proposed to be involved in the expression of high-affinity nitrate transporters of the *NRT2* gene family, such as *NRT2.1* [29], that drive nitrate uptake at low N availability. Differential phosphorylation of *NRT1.1* in dependency of the N availability and associated Ca^2+^ signaling mechanisms affect root growth through the activation of auxin transport activity and Ca^2+^-ANR1 signaling [30]. In addition, plant hormones are known to play an important role in root architecture formation [31]. Cytokinin balances the division and differentiation of stem cells [32] and its concentration in the root tip (together with auxin) defines the size of the meristem [33].

RSA was shown to be highly heritable in wheat [34,35,36,37] and several studies, investigating RSA under contrasting nitrogen levels, revealed significant differences among genotypes and between N treatments, as well as significant genotype x N treatment interactions [12,36,37] and correlations with agronomic traits such as maturity and yield [12,34,35,38]. To gain an understanding of this genetic variation and its underlying regulation of RSA, quantitative trait loci (QTL) studies are an effective tool. In recent years, many QTL for RSA traits under N deficiency have already been characterized in wheat [34,36,37,38]. 

To increase the power of QTL detection by a genome-wide association study (GWAS), the concept of multi-parent advanced generation intercross (MAGIC) populations is a valuable tool. MAGIC populations, with their increased number of founders, combine the statistical power of bi-parental populations with the genetic diversity of association panels. In wheat, a number of MAGIC populations have already been successfully used [39,40,41]. To circumvent the limitation of bi-allelic SNPs in a MAGIC population, two or more SNPs in strong linkage disequilibrium (LD) can be combined into a haploblock [42]. This allows to trace back a detected QTL effect to the parent that inherited it. Haplotype-based GWAS has already proven its ability to detect both major QTL and small-effect QTL in several studies [43,44,45]. Within the frame of this study, the WM-800 population (a MAGIC population derived from eight European winter wheat varieties) was utilized, which was shown to be a valuable mapping population for different agronomic traits [46,47,48].

To investigate the genetic regulation of RSA traits and their plasticity to N availability, a subset of 350 lines of the winter wheat MAGIC population WM-800 was non-destructively phenotyped in a germination paper-based high-throughput phenotyping system called GrowScreen-PaGe [24]. Fourteen root and shoot traits were phenotyped at three time points under two contrasting N treatments to quantify variation within the WM-800 population and variation between the N treatments. Finally, we conducted a haplotype-based GWAS to answer the question whether the genetic control of those traits depends on N availability and whether it differs between time points. 

## 2. Results and Discussion

### 2.1. WM-800 Shows Broad Variation for Root Traits

Phenotyping in the GrowScreen-PaGe system has allowed a dynamic analysis of root system growth in the winter wheat MAGIC population WM-800 under two different N treatments. The soil-free setup enabled phenotyping root systems of a large set of genotypes in a high-throughput process and provided high resolution time series data for a broad range of traits describing early root and shoot development as a function of N availability. At the same time, it must be considered that root growth and NUpE in the field is affected by a wide range of interaction effects between the root system and the soil and its microbiome. Those effects can only be represented to a limited extent in such a phenotyping system.

In total, 14 traits were examined (Table 1), which form four different trait groups and can thus comprehensively represent the development of both the root system and the shoot of the seedlings. Seminal root length (SRL) and lateral root length (LRL) consider root length, while branching angle lateral (BAL), seminal root angle (SRA) and root system radius angle left (RRL) and right (RRR) describe the angle of root growth. Root system depth (RSD), root system width (RSW) and convex hull area (CHA) are composed of root system length and angle. Finally, harvest parameters’ root dry weight (RDW), shoot dry weight (SDW), root to shoot ratio (R:S), SPAD (measurement for leaf chlorophyll content) and leaf length (LLE) were recorded 12 days after transplantation (DAT).

Significant differences were observed between genotypes and between treatments for most traits and time points, as well as significant genotype × treatment interaction effects (Table 2). An exception is LRL at 3 DAT, where no significant genotype, treatment or genotype × treatment interaction effects were observed because lateral roots had not yet been formed. Therefore, also the branching angle of the lateral roots (BAL) could not be determined 3 DAT. A broad variation in phenotypes of the analyzed 350 WM-800 lines, higher than in the founders and check varieties, was observed for all traits (Figure S1). This is an indication for transgressive segregation in the WM-800 population, which is beneficial in later genetic analysis and selection in plant breeding. The phenomenon of transgressive segregation for root traits has also been described in other wheat studies [22,49]. Studies related to plant height, yield and yield parameters in the WM-800 population already demonstrated the broad variation within this population and its power to map new QTL affecting a variety of important agronomic traits [46,47,48].

The coefficient of variation (CV) ranged between 6.67% for SPAD (N+, 12 DAT) and 95.69% for LRL (N-, 3 DAT). The root length traits and traits composed of root length and root angle, with exception of SRL, showed overall higher CV than the root angle and harvest traits. The exceptional high CV for LRL might be explained by the origin of development of lateral roots in comparison with seminal roots. Lateral roots are developed post-embryonic and thus might be more strongly affected by environmental factors [50,51].

Repeatability (Rep) varied widely among the different traits and treatments, with RSA traits except SRL and RDW showing low Rep at the last time point (12 DAT). RSD, RSW and CHA showed a high Rep in both treatments 3 and 7 DAT, which ranged from 48.34% (CHA N+ 7 DAT) to 72.97% (RSW N-3 DAT). For the other traits, the Rep under N- ranged from 11.10% (LRL 3 DAT) to 60.57% (SRA 7 DAT), while under N+ it ranged from 12.02% (RRR 12 DAT) to 61.88% (SRL 3 DAT).

**Figure 1:**
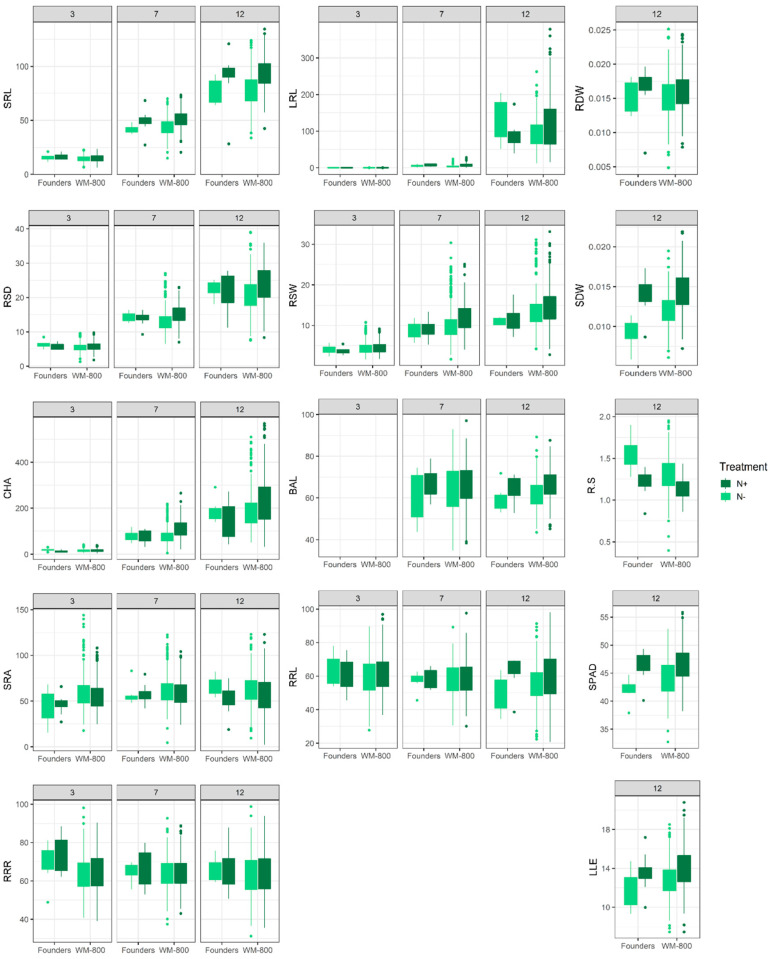
Box Whisker plots indicating trait progression over 12 days after transplanting to GrowScreen-PaGe phenotyping system per N treatment for founders and population WM-800. Trait abbreviations are indicated in Table 1.

For 19 out of 31 traits by time point combinations the mean phenotype values were significantly lower under N− compared with the N+ treatment (Figure 1 and Table S5), indicating that N deficiency had a negative effect on the root and shoot development. The only exceptions from this rule were observed for R:S at 12 DAT and SRA at all time points, where higher values under N− were observed. Wang et al. [52] and Guo et al. [53] observed similar responses of plant growth and chlorophyll content studied under contrasting N treatments. However, they also observed a significantly increased root growth under N deficiency, which was not observed in the experiment conducted here. Studies showed that nitrate stimulates not only lateral root growth [54] but also primary root growth through an increased cell division and elongation in *Arabidopsis* [55]. Since N is supplied in the form of nitrate in this experiment, this might explain the observations of longer roots under N+ compared with N−. A study in barley described the phenomenon of root growth promotion under high N supply, in case this N supply is concentrated in a specific zone and not evenly distributed [56]. Since the plants in our experiment were supplied via a nutrient solution at the bottom of the growth paper, this might also explain the observed trends. The improved root growth under N+ might also have been caused by optimal nitrate availability in the N+ treatment.

Over the course of the experiment, the values for traits related to root length and the combination of root length and angle (SRL, LRL, RSD, RSW, CHA) increased, while the traits related to root system angle changed little over time and showed no upward or downward trend. 

#### Correlations Indicate N Dependent Genotypic Plasticity

**Figure 2:**
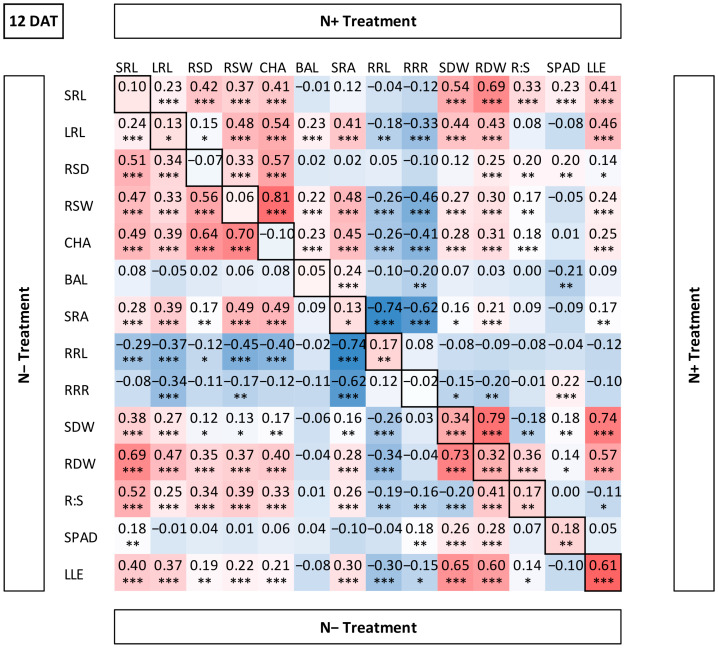
Pearson’s correlation table for 14 traits. Upper and lower triangle represent correlation coefficients (r) under N- and N+ treatments, respectively. Trait auto-correlations between both N treatments are indicated in the diagonal. Red and blue colors indicate positive and negative correlations, respectively. Trait abbreviations are indicated in Table 1. Significance levels: * *p* < 0.05, ** *p* < 0.01, *** *p* < 0.001.

Over the three phenotyping time points, correlations between traits remained rather stable (Figure S2). The traits based on root length and root length x angle (SRL, LRL, RSD, RSW, CHA) formed a block of significant positive correlations similarly in both N treatments (Figure 2). The strong positive correlations between root traits have also been observed in other studies [22,49] and might suggest that they are under shared control.

Correlations of LRL with the other traits were close to zero at 3 DAT (Figure S2), because lateral roots just started to form. Furthermore, RDW showed a strong and significant positive correlation with SDW and LLE under both treatments, suggesting a dependency of root and shoot growth and biomass accumulation. On the other hand, RDW was just moderately correlated with the two root length parameters SRL and LRL, suggesting that there is a second parameter next to root length determining the root biomass, such as root diameter, that showed significant genotypic differences in another study investigating the WM-800 population [48]. The SPAD value was positively but only weakly correlated with SRL, RDW and SDW in both treatments. 

In contrast, the auto-correlations of the traits between the two N treatments were rather low, except for LLE (r = 0.61, 12 DAT), indicating that the tested wheat genotypes developed roots as well as shoots differently under the two N treatments. This plasticity of the root system in response to environmental conditions has been widely characterized in the literature [57,58].

### 2.2. GWAS Reveals a Different Genetic Control of Root Traits under Both N Treatments

GWAS was performed separately for the two N treatments, which allowed us to investigate the genetic regulation of NUE depending on N availability. A total of 102 significant marker–trait associations (Table S6), grouped into 64 QTL, were detected for 12 of the 14 traits (Table 3). No significant QTL were found for the traits BAL and RDW. The detected QTL were distributed equally over all three wheat genomes with 24, 22 and 28 on genomes A, B and D, respectively.

A total of 24 of the QTL were N− specific, 36 N+ specific and only 2 QTL were significant under both N treatments. The finding that the traits in the two N treatments were largely regulated by different QTL suggests that selection for NUE under N deficiency is more beneficial than passive selection under standard N availability. Studies in maize and wheat could confirm the advantageousness of direct selection for N efficiency [59,60]. These individual QTL explained between 4.83% (LLE N+ 12 DAT) and 21.03% (LRL N− 7 DAT) of the genotypic variance (R^2^) (Table 4); the overall GWAS models could explain between 19.28% (SRL N− 3 DAT) and 57.53% (LLE N+ 12 DAT) of the genotypic variance per trait, treatment and time point. The R^2^ values were on average slightly higher under N+ than under N−. 

#### 2.2.1. Thirteen QTL Hotspots Showed Pleiotropic Effects on Multiple Traits

The GWAS analysis revealed 13 QTL hotspot regions of either equal or genetically linked HBs or singular SNPs having pleiotropic effects on two or more traits simultaneously. These QTL hotspots could be detected within one treatment as well as across treatments and affected between two and five traits. The five N non-specific, four N− specific and four N+ specific QTL hotspots are discussed in more detail below, complemented by the discussion about further interesting QTL regions.

##### Effects of the *Rht* Genes *Rht-B1* and *Rht-D1* and Further N Non-Specific QTL Hotspots

The GWAS revealed two QTL hotspots on chromosomes 4B and 4D associated with the well-known reduced height (*Rht*) genes *Rht-B1* and *Rht-D1*. These QTL showed effects exclusively on aboveground traits and not on root traits. While some studies, just as in this study, found no significant effects of the semi-dwarf variants of *Rht-B1* and *Rht-D1* on root traits [48,63,64], Li et al. detected significant effects on root length of a QTL mapped to *Rht-B1* [65].

The semi-dwarf phenotypes of the *Rht* genes, *Rht-B1b* and *Rht-D1b,* are a result of a mutation in the N-terminal DELLA domain of *Rht-B1* and *Rht-D1*, which prevents the two proteins being repressed by gibberellic acid (GA), leading to a reduced plant growth [66]. The founders Tobak and Safari carry the semi-dwarf allele *Rht-B1b*, while the founders Patras, Linus, JB Asano and Julius carry the semi-dwarf allele *Rht-D1b*. The founders Meister and Bernstein carry the long straw alleles at both loci.

The QTL hotspot on chromosome 4B, associated with *Rht-B1*, showed a significant effect on LLE in both N treatments with R^2^ values of 10.13 and 13.17% under N− and N+, respectively. The HT derived from Tobak and Safari reduced LLE by 10.21 and 13.19% under N− and N+, respectively.

The QTL hotspot on 4D, associated with *Rht-D1*, affected LLE across treatments, SDW under N+ and SPAD under N− at 12 DAT. For the founders Patras, Linus, JB Asano and Julius carrying the semi-dwarf allele *Rht-D1b* LLE was reduced by 11.13% under N− and 12.33% under N+, SDW under N+ was reduced by 9.45% and SPAD value under N− was increased by 4.11%. The R^2^ values ranged from 6.82% (SPAD N−) to 15.75% (LLE N+). The N non-specific genetic regulation of LLE by *Rht-B1* and *Rht-D1* is also reflected in the high auto-correlation between the two N treatments.

A next hotspot was localized on chromosome 2A. It significantly affected LRL (N- 7 DAT) and R:S (N+ 12 DAT) and could explain 21.03 and 8.90% of the variance, respectively. Another hotspot was located on 2D and consisted of two QTL in close genetic distance. They affected SRL (N+ 7 DAT) and RSW (N− 7 DAT) and explained 8.54 and 11.96% of the variance, respectively. For both hotspots on chromosome 2A and 2D a candidate gene has not yet been identified.

A last N non-specific QTL hotspot was located on chromosome 7A and affected RSW under N+ at 3 DAT and RRL under N− at 3 DAT with a relative effect of 18.17 and 12.74% for the QTL allele derived from Meister, Linus and Julius, respectively. A candidate gene has not yet been identified.

##### N− Specific QTL Hotspots

The hotspot that affected the most traits under N- was located on chromosome 5A at 688–691 Mbp. This HB (Chr5A_HB137, see Table S6) affected SRL (12 DAT), RSD (7 DAT), RSW (7 DAT), SRA (7 DAT) and RRL (7 DAT). Five different HTs could be differentiated, but only the HT of the founder JB Asano was significant and showed strong relative effects ranging from −16.41 to 50.05% on RRL and RSW, respectively. A candidate gene is *NRT1.1* (IWGSC RefSeq v1.1: TraesCS5A02G537100.1 [61]) in close proximity to the detected HB. *NRT1.1* belongs to the gene family *NRT1*, which codes for low-affinity nitrate transporters, acting under high nitrate concentration. However, in *Arabidopsis*, *NRT1.1* is known to change its function under low nitrate availability by being phosphorylated, acting as a nitrate sensor instead [30]. An expression study in wheat demonstrated a higher expression of *NRT1.1* under low nitrate availability [53]. The cascade downstream of the phosphorylated *NRT1.1* activates phosphatidylinositol, resulting in a Ca^2+^ influx that then changes the expression of transcription factors and genes involved in nitrate transport (high-affinity transporters of the gene family *NRT2*) and assimilation. The activated high-affinity transporters in the end affect lateral root initiation and growth [30].

A QTL hotspot on chromosome 3B (492–497Mbp) showed significant effects on RSD (QRSD3_N-_WM-800_3B) and RSW (QRSW3_N-_WM-800_3B) at 3 DAT under N-. The HT derived by the founders Patras, Tobak and Safari reduced RSD and RSW by 25.67 and 51.85% with an R^2^ value of 9.00 and 17.74%, respectively. Within this HB, a candidate gene is located coding for an SKP1-like protein (IWGSC RefSeq 1.1: TraesCS3B02G308600.1 [61]). These proteins have been described to affect seed germination and seedling growth in *Arabidopsis* [67,68] and are upregulated under abiotic stress [67]. SKP1 is a S-phase kinase-associated protein 1 and a component of the SCF-type E3 ligase, where it regulates the signaling pathways of several phytohormones [68]. A study in wheat found that a *SKP1* gene was highly expressed in the elongation zone of young roots and an overexpression of *TSK1* in *Arabidopsis* mutants resulted in changes in the auxin response and auxin-related root phenotypes [69].

One further hotspot that was significant exclusively under N- was located on chromosome 4A and affected RSD and RSW at 3 DAT. The significant HT was carried only by the founder Safari, increasing RSD and RSW by 18.71 and 27.54%, respectively. In direct proximity to this hotspot, a candidate gene is located (IWGSC RefSeq v1.1: TraesCS4A02G312300.1 [61]) that is highly expressed in root tissue [70,71] and codes for a calcium-dependent lipid-binding domain protein. Phospholipids, precisely the phosphoinositide/phospholipase C (PI/PLC) system, and Ca^2+^ are both known to be involved in stress signaling in plants [72,73]. Several studies described the role of calcium-dependent lipid-binding proteins in abiotic stress response [74,75]. A study in rice proposed that the calcium-dependent lipid-binding domain protein might affect Ca^2+^ influx by binding to the phospholipid. An overexpression of the coding gene led to a more drought stress resistant phenotype with longer roots [75]. This cascade of PI/PLC activation and subsequent Ca^2+^ influx also play a key role in the NO_3_^−^ dependent changes in the expression of transcription factors and genes involved in nitrate transport and nitrate assimilation downstream of *NRT1.1*. *NRT1.1* is a candidate gene we already discussed for a previous N- specific hotspot on 5A that, in the end, affects lateral root initiation and growth [30]. 

The last N- specific hotspot was located on chromosome 6B between 25.09 and 33.72 Mbp and affected RSW (3 DAT), SRA (7 DAT), CHA (7 DAT) and RRL (7 DAT). The relative effect of the HT derived from Meister ranged from 9.87 to 35.55% for RRL (7 DAT) and CHA (7 DAT), respectively. In close distance to the significant HB are 11 genes coding for high affinity nitrate transporters, of which six (IWGSC RefSeq v1.1: TraesCS6B02G044000.1, TraesCS6B02G044100.1, TraesCS6B02G044200.1, TraesCS6B02G044300.1, TraesCS6B02G044400.1, TraesCS6B02G044500.1 [61]) are expressed in roots in the vegetative stage [70,71]. These high-affinity nitrate transporters of the gene family *NRT2* have been proposed to be involved in root growth downstream of the nitrate transporter NRT1.1 [30]. One of these genes is *TaNRT2.1* (IWGSC RefSeq v1.1: TraesCS6B02G044300.1 [61]) that increased N influx and root growth in wheat when overexpressed and also showed increased grain yield and N accumulation [76], making it a potential candidate for NUE breeding.

##### N+ Specific QTL Hotspots

We found that an N+ specific hotspot on chromosome 1B had a significant effect on RSW (QRSW3_N+_WM-800_1B) and CHA (QCHA3_N+_WM-800_1B) at 3 DAT. The HT, derived from the founders JB Asano, Meister, Patras, Safari and Tobak, increased RSW and CHA by 23.73 and 25.00% with an R^2^ value of 10.56 and 8.08%, respectively. A potential candidate gene is a gene coding for a mei2-like 2 protein (IWGSC RefSeq v1.1: TraesCS1B02G164600.1 [61]) at 287.5 Mbp. The *mei2* gene family was first described for its role in meiosis, but newer studies have observed a role in seedling development and meristem activity as well [77]. They observed an alternated root phenotype, suggesting alternated meristem activity for *mei2* mutants and an expression of *mei2*-genes in the roots, which is consistent with the expression pattern for our candidate gene TraesCS1B02G164600.1 [70,71]. A potential explanation for the QTL just being significant under N+ is that the N deficiency masks the effect of this QTL under N-.

Another N+ specific QTL hotspot on chromosome 2D had strong significant effects on CHA at 3 DAT and on RSW at 7 DAT. The founder Linus carried the significant HT that increased CHA and RSW by 44.80 and 28.14%, respectively. Two further QTL hotspots were located on chromosomes 6B and 6D. The QTL on 6B affected LRL at 3 DAT and RSD at 7 DAT and explained 14.36 and 7.80% of the variation, respectively, while the QTL on 6D affected R:S and RSW at 12 DAT with an R^2^ value of 6.05 and 12.74%, respectively. It remains open which genes are responsible for the observed effects on 2D, 6B and 6D. 

Additional QTL of interest showing a significant effect on only one trait are sorted by trait categories and discussed in the following.

#### 2.2.2. Number of Detected QTL Suggests High Plasticity of Lateral Roots to Environmental Influences

Eight QTL have been detected for the root length related traits SRL, while only four for LRL. The QTL were distributed evenly over time points and N treatments. The significantly higher amount of detected QTL for SRL than for LRL might be explained by the fact that seminal roots of plants are established during embryogenesis while lateral roots are established post-embryonically [51] and, thus, might be affected by environmental influences stronger than seminal roots.

##### Seminal Root Length (SRL)

In addition to the two QTL on chromosome 2D and 5A, which have already been discussed as hotspots, six further QTL were detected for SRL. One of these QTL was located on chromosome 2D and the significant HT, derived from Patras, JB Asano, Tobak and Julius, increased SRL under N- at 12 DAT by 12.01%. Two candidate genes within the HB that are highly expressed just in root tissue [70,71] code for ABC transporters of the subfamily B (IWGSC RefSeq v1.1: TraesCS2D02G010400.1 and TraesCS2D02G010500.1 [61]). The role of ABC subfamily B transporters in root development has been described in multiple studies [78,79]. ABC transporters are known to be located in the plasma membrane and function in auxin transport and, thus, auxin regulated development [80,81]. Variation in the auxin transport could explain the observed effects on SRL.

The next QTL on chromosome 7B affected SRL (QSRL3_N+_WM-800_7B) under N+ at 3 DAT. The significant HT is present in the founders JB Asano, Julius, Meister, Patras and Tobak and showed a relative effect of 15.77%. Close to this QTL a gene is located coding for a glycosyltransferase (IWGSC RefSeq v1.1: TraesCS7B02G340100.1 [60]) with high expression in root tissue [70,71]. Glycosyltransferases are described as a required component for normal cell-cycle regulation and a reduced expression can result in an extended cell cycle and reduced growth [82]. This observation can be explained, most likely, by the effect of glycosyltransferases on the expression of many flavonoids, which function as auxin transport inhibitors [83]. Natural allelic variation might, thus, explain the observed effect on root growth in our study. 

##### Lateral Root Length (LRL)

For LRL, only four QTL were detected, of which the two on chromosome 2A and 6B also showed an effect on other traits. One N− specific QTL was detected on 2A at 7 DAT, which is consistent with the low repeatability under N− at the two other time points. The very high CV for LRL, which decreased slightly over time, may explain the high relative effects.

The QTL on chromosome 3D was detected under N+ at 12 DAT and the significant HT, carried by Safari, increased LRL by 59.69% and could explain 8.87% of the variation. A potential candidate gene upstream of this QTL (IWGSC RefSeq v1.1: TraesCS3D02G474800.1 [61]) is highly expressed specifically in roots [70,71] and encodes expansin, a cell wall-loosening protein [84] involved in numerous developmental processes in shoots and roots during which cell wall modifications occur [85,86,87]. A study in *Arabidopsis* proved that expansin plays a role in lateral root initiation by acting in radial pericycle cell expansion [88].

#### 2.2.3. The Traits Describing the Spatial Distribution of Root Systems Are Mainly Controlled by QTL Hotspots

RSD, RSW and CHA reflect the area encompassed by the root system of the plants. A total of 26 QTL were detected, equally distributed across both N treatments. Among them, only eight QTL had an effect on one trait only; the others were all assigned to one of the before-mentioned hotspots.

##### Root System Depth (RSD)

Four of the five detected QTL were part of a hotspot and only one QTL on chromosome 3A had an effect exclusively on RSD. The significant HT, which originated from the founders JB Asano, Bernstein and Julius, had a relative QTL effect of −13.67% and an R^2^ value of 6.99% under N+ at 3 DAT, respectively. A candidate gene could not be identified yet.

##### Root System Width (RSW)

Twelve QTL showed a significant effect on RSW, nine of which were part of a QTL hotspot. One of the three remaining QTL on chromosome 2B had a significant effect of −18.91% with an R^2^ value of 7.98% under N+ at 7 DAT. The HT with the significant effect was derived from the founders Bernstein, Linus, Meister, Safari and Tobak. In close proximity to this hotspot is the candidate gene *TaWOX11b* [89] (IWGSC RefSeq v1.1: TraesCS2B02G117900.1 [61]), coding for a WUSCHEL-related homeobox transcription factor. *TaWOX11* showed an increased expression in roots in the vegetative state [70,71,89], suggesting a role in root development that would explain the observed QTL effect. A putative rice orthologue of *TaWOX11*, *OsWOX11*, was described to be involved in the regulation of crown root development [90] by affecting the expression of auxin- and cytokinin-responsive genes.

##### Convex Hull Area (CHA)

In addition to the two QTL already discussed as hotspots, six additional QTL affected CHA. These six QTL showed strong relative effects between −43.90% (QCHA7_N+_WM-800_7D) and −38.79% (QCHA12_N+_WM-800_7A). The QTL on 7A, affecting CHA under N+ at 12 DAT, is surrounded by four *NRT1* genes and four *PTR* genes. The candidate gene (IWGSC RefSeq v1.1: TraesCS7A02G054000.2 [61]) is coding for a NRT1/PTR family 2.3-like protein that is highly expressed specifically in root tissue [70,71]. Transporters of the *NRT1* gene family are low-affinity transporters, acting under sufficient N availability, which explains the QTL detection solely under N+. The *Arabidopsis* orthologue *NPF2.3* is expressed in root pericycle cells and the transporter itself is located in the plasma membrane. Since N is known to be an important compound in the regulation of root growth [18], *NPF2.3* is a plausible candidate gene for the detected QTL.

On chromosome 7D, a QTL was located that had a significant effect on CHA under N+ at 7 DAT, explaining 9.32% of the genotypic variation. The significant HT is solely present in the founder Meister and increased CHA by 43.90%. As such, this QTL showed one of the strongest relative effects of this study. Next to the significant HB, two genes are located (IWGSC RefSeq v1.1: TraesCS7D02G000700.1 and TraesCS7D02G000800.1 [61]) that are highly expressed, especially in root tissue [70,71], and coding for a receptor-like protein kinase and a lectin-receptor kinase, respectively. Both belong to the Receptor-Like Kinase (RLK) protein family that forms a group of signaling molecules on the surface of cells, which are responsible for cell-to-cell communication and are, among others, described to control differentiation of the root meristem [91]. Lectin receptor kinase is one class of the RLK protein family and is involved in the regulation of plant hormones such as abscisic acid [92], which is known to affect root growth [93,94].

#### 2.2.4. QTL for Root System Angle-Related Traits Are Mainly Located in Close Proximity to Nitrate Transporter Coding Genes

For the root system angle traits, with the exception of BAL, 13 QTL were identified that could explain between 6.20 and 11.91% of the variance and had relative effects ranging from −22.09 to 30.04%. It is striking that five of the six N- specific QTL were located in hotspots, whereas this was not true for any N+ specific QTL. Four of these five QTL were located in close proximity to nitrate transporters, which, by their function of N sensing [30], presumably influence the root angle so that the roots can reach N rich regions. 

In general, root system angle traits are little studied in wheat so far. *VRN1* is one of the few genes in wheat whose effect on root system angle has already been characterized [95]. Furthermore, genes affecting gravitropism are strong candidate genes for root system angle traits [96]. However, the QTL detected in this study are not localized near such genes.

#### 2.2.5. The Majority of the QTL Detected for Harvest-Related Traits Were N+ Specific

At the third phenotyping time point (12 DAT), the plants were harvested. For the parameters SDW, R:S, SPAD and LLE, 13 QTL were detected, four under N− and nine under N+. As mentioned above, for LLE, significant QTL linked to the *Rht* genes *Rht-B1* and *Rht-D1* on chromosome 4B and 4D were detected in both treatments; all remaining QTL were N-treatment specific. In contrast to other studies [97,98], no QTL were detected for the trait RDW in this study. Only one QTL, part of the QTL hotspot linked to *Rht-D1*, was detected for SDW.

##### Root to Shoot Ratio (R:S)

Two of the four QTL were part of the QTL hotspots on 2A and 6D. The remaining two QTL on 5B and 6B were detected under N− and N+ and explained 7.61 and 14.92% of the variance, respectively. Candidate genes could not be identified yet.

##### SPAD

In addition to the QTL hotspot on chromosome 4D, another QTL on 4D at 211 Mbp showed a significant relative effect of 4.33% and explained 8.28% of the variance under N+. Upstream of this QTL are three genes coding for photosystem II reaction center proteins (IWGSC RefSeq v1.1: TraesCS4D02G155100.1, TraesCS4D02G155200.1 and TraesCS4D02G155500.1 [61]. The SPAD value provides information about the chlorophyll content of the plant and chlorophyll is a central component of the photosystem II reaction center, which renders these three genes potential candidates to explain the observed effect. 

One further QTL on chromosome 7A affected SPAD under N+ at 12 DAT. The significant HT derived from Meister, Linus and Safari reduced SPAD by 4.30%. In close genetic distance are two genes coding for peptide transporters (PTR) (IWGSC RefSeq v1.1: TraesCS7A02G381500.1 and TraesCS7A02G381800.1 [61]). PTRs transport peptides within the plant and, consequently, play a role in the distribution of N within the plant [99]. Genetic variation in a PTR coding gene could explain differences in SPAD values, which are closely linked to the N content in the leaves through differences in the distribution of N within the plant. 

##### Leaf Length (LLE)

Only one further QTL on chromosome 3D affected LLE under N+ at 12 DAT other than the two hotspots linked to *Rht-B1* and *Rht-D1*. Tobak is the only founder carrying the significant HT, which reduced LLE by 10.12%. A candidate gene (IWGSC RefSeq v1.1: TraesCS3D02G358900.2 [61]) codes for a O-fucosyltransferase, which is described in *Arabidopsis* as mono-O-fucosylate DELLA, enhancing its activity [100]. DELLA is a negative regulator of gibberellin signaling and, thus, of gibberellin-responsive growth [101], which could explain the observed effect on LLE. 

## 3. Materials and Methods

### 3.1. Plant Material

Due to capacity limitations of the phenotyping platform, a randomly selected subset of 350 lines of the WM-800 (a winter wheat MAGIC population) was used. Eight modern German elite varieties (released between 2008 and 2017; Table S1) were crossed according to a crossing scheme adapted from Cavanagh et al., [102]. In the F_4:6_ generation, 1323 recombinant inbred lines (RILs) were randomly selected in a first step. In a second step, all double-dwarf lines, carrying *Rht-B1b* and *RhtD1b*, were excluded and the final WM-800, consisting of 800 lines in generation F_4:7,_ was established. More details on the WM-800 population can be found in Sannemann et al., [47].

### 3.2. Plant Cultivation and Experimental Set-Up

**Figure 3:**
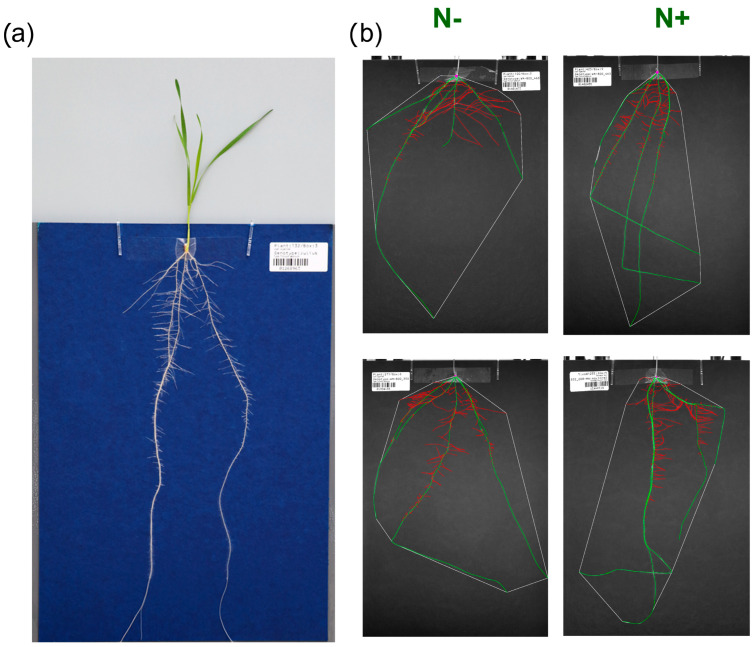
(**a**) Image of a wheat plant mounted on the blue germination paper, (**b**) images of the root systems under the two contrasting N treatments at 12 days after transplantation processed by the workflow of GrowScreen-PaGe platform. Seminal roots are labeled in green and lateral roots in red.

Plants were grown and phenotyped using the GrowScreen-PaGe phenotyping platform (Figure 3) [24,103], specializing in the non-invasive study of root morphology. 

Seeds of the 350 WM-800 lines, eight founders and four check varieties (Bonanza, Elixer, Genius, RGT Reform) were pre-germinated between two moist filter papers at 22 °C/18 °C (day/night) temperature in a darkened petri dish for 24h. 

To prevent fungal infection, seeds were treated in advance with the fungicide prothioconazole (33 mL/100 kg seed). For each genotype, 40 seeds were pre-germinated and, after 24h hours, twelve uniformly germinated seedlings were selected and transferred, seminal root facing downwards, to the germination paper (size: 37 cm × 25 cm; smooth dark blue 194 grade paper; Ahlstrom Germany GmbH, Bärenstein, Germany) and attached with transparent self-adhesive tape (Opsite Flexifix, 7478029, Smith & Nephew GmbH, Hamburg, Germany). Using transparent shirt clips (shirt clip 38 mm, Georg Scharf GmbH, Balingen, Germany), a seedling on its germination paper was attached to each PVC plate (RAL 7011, Max Wirth GmbH, Braunschweig, Germany) on the front and back side [24].

The germination paper was then sprayed until saturation with a modified Hoagland nutrient solution with two different nitrogen concentrations for the different nitrogen treatments with N− = 0 µM Ca(NO_3_)_2_ × 4H_2_O and N+ = 2000 µM Ca(NO_3_)_2_ × 4H_2_O (for details see Table S2). The PVC plates were positioned vertically with the attached plants in opaque PVC containers, with a maximum of 25 plates spaced 2 cm apart. To further shield the roots from light, adjustable black strip brushes (Mink GmbH & Co. KG, Göppingen, Germany) were attached in front of the top opening of the box through which the shoot grows (for details see Pariyar et al., [24]). The containers were each filled with 12 L of modified Hoagland nutrient solution so that the bottom 5 cm of the germination paper was submerged in the solution. The nutrient solution was replaced seven days after transplanting from the petri dishes.

A total of 350 lines and eight parents were studied in two nitrogen treatments, with six replicates each arranged in a completely randomized block design. For capacity reasons, the plants were divided into eight experimental runs. Each experimental run consisted of two blocks with six boxes each (three N- and three N+ boxes). In each run, 46 lines (WM-800 and founder) and four checks were tested. The six repetitions of the 46 lines were divided among the six boxes. The four checks were included in each box and randomly placed. Plants were grown in a controlled growth chamber at 22 °C/18 °C (day/night) air temperature, 12 h/12 h light/dark, 60% relative air humidity and ~150 μmol m^−2^ s^−1^ photosynthetically active radiation (PAR) at leaf level (Johnson Controls Systems & Service GmbH, Leipzig, Germany).

### 3.3. Phenotypic Data

High-resolution images (74 μm per pixel) of the root system were taken 3, 7 and 12 days after transplanting (DAT) to the germination paper using 16 MP cameras (IPX-16M3-G, monochrome, Imperx Inc.; combined with Zeiss Planar T 2.0/50 ZF-I lens) in a mobile imaging box, described in Gioia et al., [103]. At the last phenotyping time point, plants were harvested after imaging was finished. Leaf length and SPAD value of the plants were measured manually before roots and shoots were separated above the seed. Dry weights of roots and shoots were determined after drying plant materials at 65 °C in an oven until constant weight was reached.

For the analysis of the images, the workflow of the GrowScreen-PaGe system was used, which first segmented the roots from the background and labeled seminal and lateral roots based on their origin (from the grain or from the seminal roots, respectively) [24]. The segmentation and the labeling were manually checked in a second step and corrected if necessary. In the final step, the images were processed to quantify a total of ten root system architectural traits. Five root- and shoot-related harvest parameters, collected at 12 DAT, completed the overall picture (Table 1 and Table S3). In addition, tags were set (such as “not germinated”, “damaged root”, scored as true or false, Table S4) to assess the data quality and were subsequently used for data cleaning. 

The 14 root- and shoot-related traits can be summarized into four trait groups. Seminal root length (SRL) and lateral root length (LRL) consider root length, while branching angle lateral (BAL), seminal root angle (SRA) and root system radius angle left (RRL) and right (RRR) describe the angle of root growth. Root system depth (RSD), root system width (RSW) and convex hull area (CHA) are composed of root system length and angle. Finally, harvest parameters’ root dry weight (RDW), shoot dry weight (SDW), root to shoot ratio (R:S), SPAD and leaf length (LLE) were recorded 12 days after transplantation.

### 3.4. Statistical Analysis

Statistical analysis was performed with the software RStudio [104].

A first analysis of variance (ANOVA), modeling genotype, treatment and experimental run (batch) as fixed effects revealed significant batch effects, which is why we have normalized the data. In a first step, the least squares means (LSmeans) of the four check varieties across both N treatments and per batch were calculated using the package “emmeans” [105] and each WM-800 line value was divided by the mean LSmean of the checks of the corresponding batch. A mean LSmean was then calculated for the checks across the batches and both treatments, by which each normalized WM-800 value was multiplied. A second ANOVA after normalization revealed no more significant batch effects. Afterwards, ANOVA was recalculated to test for genotype and treatment effects and the two-way interaction effect.

In a first data cleaning step, all measurements that were scored as “true” for a tag that showed a significant effect on the trait, based on an ANOVA modeling genotype, treatment and tag as fixed effects, were discarded. In addition, outliers, where for instance the root length-related traits were close to zero (reflecting strong growth disorders), were removed.

Variance components were estimated separately for both N treatments using the package “blme” [106] with genotype modeled as a random effect. In a second step, a linear model containing genotype as fixed effect was used to estimate the standard error (SE) of the adjusted means, which, in the next step, was used for calculation of LSmeans and repeatability. Repeatability was estimated using the genetic variance and the standard error from the two previously described steps as described in the following formula [107]:

$$Rep=\;\frac{\sigma_{G}^{2}}{\sigma_{G}^{2}+{SE}^{2}}$$

where $\sigma_{G}^{2}$ is the genotypic variance and *SE*^2^ is the squared standard error of the difference between the LSmeans.

Then LSmeans for each genotype of the WM-800 over the six replications were calculated and the Spearman correlation coefficients between the traits and between the treatments were calculated using the package “psych” [108].

### 3.5. Genotypic Data

The 800 WM-800 lines, their eight founders and the four checks were genetically characterized with the Infinium 15K iSelect SNP array [47] and the 135k Affymetrix SNP array [46], both from SGS TraitGenetics (Gatersleben, Germany), using bulked DNA from 12 F_4:5_ seedlings per genotype.

All SNPs polymorphic in WM-800 have undergone a quality check (SNP calls for all founders, <5% missing calls, >5% minor allele frequency [109] and a known physical position in the wheat genome), of which 27,006 polymorphic SNPs passed. The physical positions of the SNPs, anchored to the Refseq v1.0 reference genome sequence of *Triticum aestivum* [61], were provided by SGS TraitGenetics (Gatersleben, Germany). For the SNPs of the 15K iSelect SNP array, genetic positions were provided and for the remaining SNPs, the genetic position was assigned to the wheat consensus map [62] using LD mapping as described in [110].

To perform regression analysis, the 27,006 polymorphic SNPs were transcribed into a numerical matrix based on identity by state (IBS) according to the Julius founder allele [47]. All genotypes with the same homozygous status as Julius were coded as 2, all genotypes showing a homozygous Non-Julius allele were coded as 0 and heterozygous genotypes were coded as 1. In a last step, missing genotype calls were imputed using the mean imputation approach (MNI), which replaces each missing SNP value by the mean value of the remaining genotypes [111].

### 3.6. Haplotype Building

The software Haploview 4.2 [112] was used to create a haplotype (HT) matrix for the WM-800 population, made of SNPs in high LD. For this, all SNP genotypes of the eight founders with a minor allele frequency MAF > 0.05 were used. SNP pairs with a distance of <500 kb and consecutive SNPs were combined in a haploblock (HB) if (i) at least one out of the four possible gametes was observed with a frequency of <0.01 and (ii) a strong LD of D’ = 1 was estimated between the SNP pairs. SNPs that were not included in a HB were retained as so-called “singular SNPs”.

A total of 2970 HBs with 92,734 HTs have been identified. A total of 8498 of these HTs met the quality criteria of (i) HT frequency > 5% in WM-800 and (ii) HT sequence without missing nucleotides. These HTs were supplemented with 4562 singular SNP alleles that could not be assigned to any HB. Finally, the genotype scores were coded into a 0–1 (absence–presence) matrix to enable GWAS in a multiple linear regression framework (see [113], File D).

### 3.7. GWAS

GWAS was performed using SAS 9.4 software (SAS Institute Inc., Cary, NC, USA, 2016). In the first step, all SNP alleles and HTs associated with the targeted trait were selected using a multiple linear regression model (SAS PROC GLMSELECT). This was accomplished by creating 100 repeated subsamples, each containing 80% of the lines, and selecting only the SNP alleles and HTs that improved the prediction of the remaining 20% (corresponding to the minimum mean squared error). Those SNP alleles and HTs that were selected more than once in this step were defined as potential cofactors. Potential cofactors were then used as input for the final selection of cofactors (SAS PROC GLMSELECT based on minimizing the Schwarz–Bayesian criterion) in the complete dataset. Selected cofactors were then modeled using SAS PROC REG in the background as part of a multiple linear regression model in which all SNP alleles and HTs were tested for significance. HTs and SNP alleles with a −log10 Bonferroni corrected *p* value < 0.05 were selected as significant. Thus, allele effects, R^2^ and Bonferroni corrected *p*-value were estimated as a function of the cofactors that entered the model first according to their ranking in the previous step by applying the PARTIALR2 (SEQTESTS) model option. A relative QTL effect was calculated by dividing the absolute estimated effect by the corresponding population mean. If a QTL or two or more linked QTL showed an effect on more than one trait they were classified as QTL hotspots. Note that the first four principle components (explaining more than 5%), presented in Lisker et al., 2022 [46], were included in the model in all steps mentioned above to correct for potential population structure effects.

### 3.8. Candidate Genes

For the candidate gene search, the physical positions of the HBs and singular SNPs anchored to the Refseq v1.1 reference genome sequence of *Triticum aestivum* [61] were used. The gene annotation from the IWGSC was used to identify genes either within the significant HB or upstream and downstream until reaching the neighboring HBs (complete list of all genes within the respective regions can be found in Table S7). Subsequently, to define candidate genes, these genes were screened for (i) their function, (ii) expression profiles using the wheat RNA-seq expression database of polyploid wheat (http://www.wheat-expression.com/ (accessed on 15 August 2022)) and (iii) information given in the literature. Candidate genes, for which all three criteria fit, were discussed in more detail in the text.

## 4. Conclusions

Root growth during the first days after germination provides an important foundation for plant establishment and later plant development. Both the finding of significant main effects for genotype and N treatment as well as genotype by N treatment interactions and the detection of numerous N specific QTL confirm the effectiveness of the measurement method and the dependence of the regulation of RSA traits on N availability. These findings are promising for breeding towards an increased nitrogen uptake efficiency by allowing plants to adapt well to marginal soils and to lower N input levels by an optimized root system.

The GWAS revealed 64 significant QTL. Due to the use of a haplotype-based GWAS approach, the effects could be assigned to the individual parents of the MAGIC population. Of these QTL, 24 were N− specific, 36 N+ specific and only 2 QTL were found active in both N treatments. In this context, at 13 QTL hotspots, pleiotropic effects were observed. In addition to the prominent *Rht* genes, *Rht-B1* and *Rht-D1*, candidate genes under N− were, among others, *NRT1.1* and genes involved in stress signaling and hormone regulation. In contrast, candidate genes under N+ were more associated with regulation of general growth, examples being *mei2* and *TaWOX11b*.

The clear trend that more N specific than N non-specific QTL were detected suggests the differential genetic regulation of traits as a function of N availability. This is an indication that a direct selection for tolerance of reduced N availability under N deficiency is more effective in breeding than indirect selection under standard N availability. 

To further investigate the regulation of RSA traits and the effects of RSA on yield and quality traits under differing N availability under field conditions, heterogeneous inbred families (HIFs) [114,115] can be generated easily from WM-800 lines that were heterozygous at the loci of interest in generation F_4_. The concept of HIFs allows QTL validation and fine mapping to ultimately clone and verify the responsible genes via transformation or knock out.

Deciphering the genetic regulation of RSA under N stress and integrating the trait-improving QTL alleles in modern plant breeding programs might be an important step in the selection of stress-adapted varieties that will allow to feed the human population under increasing requirements for environmental sustainability and resource protection.

## Figures and Tables

**Table 1:** List of analyzed root and shoot traits including trait name, abbreviation, description and unit.

Trait	Abbr.	DAT ^a^	Trait Group	Description	Unit
Seminal Root Length	SRL	3, 7, 12	Root length	Length of seminal roots	cm
Lateral Root Length	LRL	3, 7, 12		Length of lateral roots branched from seminal roots	cm
Root System Depth	RSD	3, 7, 12	Composition of root length and root angle	Maximal vertical depth of a root system	cm
Root System Width	RSW	3, 7, 12		Maximal horizontal distribution of a root system	cm
Convex Hull Area	CHA	3, 7, 12		Area of the convex hull that encompasses the whole root system	cm^2^
Branching Angle Laterals	BAL	7, 12	Root angle	Average branching angle between the seminal roots and branched lateral roots	°
Seminal Root Angle	SRA	3, 7, 12		Angle between outermost seminal roots measured at a distance of 5 cm from the seed	°
Root System Radius Angle Left	RRL	3, 7, 12		Angle of the outermost left seminal root to the horizontal measured at 5 cm distance from the seed	°
Root System Radius Angle Right	RRR	3, 7, 12		Angle of the outermost right seminal root to the horizontal measured at 5 cm distance from the seed	°
Root Dry Weight	RDW	12	Harvest parameters	Root dry weight after drying roots at 65 °C until constant weight was reached	g
Shoot Dry Weight	SDW	12		Shoot dry weight after drying roots at 65 °C until constant weight was reached	g
Root to Shoot Ratio	R:S	12		R:S = RDW:SDW	
Soil Plant Analysis Development (SPAD) Chlorophyll Meter	SPAD	12		SPAD value, one measurement per plant, provides information about the chlorophyll content of the plant	SPAD units
Leaf Length	LLE	12		Length of longest leaf	cm

^a^ Day after transplanting the germinated seeds to the germination paper.

**Table 2:** Descriptive statistics of 14 traits measured in population WM-800 under two N treatments at three time points. Trait abbreviations are indicated in Table 1.

Trait	T ^b^	CV ^c^			Rep ^d^			P(G) ^e^			P(T) ^f^			P(G × T) ^g^		
		3 ^a^	7 ^a^	12 ^a^	3 ^a^	7 ^a^	12 ^a^	3 ^a^	7 ^a^	12 ^a^	3 ^a^	7 ^a^	12 ^a^	3^a^	7 ^a^	12 ^a^
**SRL**	**N−**	19.91	18.32	19.85	47.04	39.28	50.70	***	***	***	*	***	***	*	***	***
	**N+**	22.51	16.24	16.37	61.88	45.03	50.75									
**LRL**	**N−**	95.69	70.47	42.95	11.10	34.01	16.40	-	***	***	-	***	***	-	***	***
	**N+**	92.02	67.79	59.29	21.29	46.60	50.37									
**RSD**	**N−**	23.87	25.33	23.60	59.21	54.88	22.92	***	***	***	-	***	*	***	***	***
	**N+**	22.43	18.38	23.77	58.70	49.05	19.20									
**RSW**	**N−**	37.69	42.01	33.96	72.97	72.59	35.59	***	***	***	-	***	-	***	***	***
	**N+**	32.97	29.62	34.74	68.07	58.37	39.41									
**CHA**	**N−**	45.21	44.53	43.45	69.96	53.98	29.69	***	***	***	-	***	*	***	***	***
	**N+**	39.92	34.47	48.17	62.98	48.34	38.17									
**BAL**	**N−**		18.65	11.52		26.97	20.91		***	***		*	***		***	**
	**N+**		15.19	10.50		30.24	34.22									
**SRA**	**N−**	31.85	28.12	29.17	54.73	60.57	32.83	***	***	***	**	**	**	***	***	***
	**N+**	28.20	24.70	35.49	44.81	53.23	28.41									
**RRL**	**N−**	19.64	17.75	21.08	47.27	60.40	25.95	***	***	***	-	**	***	***	***	*
	**N+**	18.83	16.25	23.49	36.41	44.32	23.16									
**RRR**	**N−**	15.11	12.42	18.18	32.01	32.78	31.41	***	***	***	***	-	-	***	***	***
	**N+**	15.75	12.87	18.44	44.45	41.04	12.02									
**SDW**	**N−**			16.98			42.36			***			***			***
	**N+**			16.89			54.24									
**RDW**	**N−**			18.58			45.57			***			***			***
	**N+**			17.41			55.51									
**R:S**	**N−**			17.77			68.71			***			***			***
	**N+**			10.62			36.04									
**SPAD**	**N−**			7.36			66.90			***			***			***
	**N+**			6.67			69.45									
**LLE**	**N−**			14.25			60.96			***			***			**
	**N+**			14.52			74.97									

^a^ Days after transplanting (DAT); ^b^ treatment: low nitrogen (N-), high nitrogen (N+); ^c^ coefficient of variation (%), red and blue colors indicate high and low values, respectively; ^d^ repeatability (%); ^e^ significant differences between genotypes across N treatments: *** *p* < 0.001; ^f^ significant differences between N treatments: * *p* < 0.05, ** *p* < 0.01, *** *p* < 0.001; ^g^ significant effects of the genotype x treatment interaction: * *p* < 0.05, ** *p* < 0.01, *** *p* < 0.001.

**Table 3:** Number of significant QTL per trait, treatment and time point (DAT), detected specifically under N- and N+, respectively, and explained genotypic variance (R^2^, in %). Trait abbreviations are indicated in Table 1. Red and blue colors indicate high and low values, respectively.

Trait	Treat	3 DAT		7 DAT		12 DAT	
		No. QTL	R^2^ (%)	No. QTL	R^2^ (%)	No. QTL	R^2^ (%)
SRL	N−	1	19.28	0	11.60	2	22.89
	N+	1	29.59	2	28.16	2	33.48
LRL	N−	0	23.91	1	26.40	0	4.43
	N+	2	46.14	0	1.17	1	21.58
RSD	N−	2	23.97	1	20.58	0	19.49
	N+	1	29.69	1	26.20	0	20.05
RSW	N−	3	38.87	2	31.22	0	26.38
	N+	4	42.46	2	29.39	1	37.40
CHA	N−	2	42.77	1	24.11	1	19.29
	N+	3	41.34	1	37.92	1	35.54
BAL	N−			0	16.18	0	11.09
	N+			0	11.46	0	22.49
SRA	N−	0	22.78	2	28.29	0	18.83
	N+	1	28.49	1	28.23	1	31.24
RRL	N−	1	19.87	2	26.15	0	22.72
	N+	0	14.11	1	20.92	0	18.08
RRR	N−	1	29.95	0	25.58	0	16.80
	N+	1	38.14	2	31.62	0	21.15
SDW	N−	-		-		0	24.96
	N+	-		-		1	28.79
RDW	N−	-		-		0	24.00
	N+	-		-		0	24.48
R:S	N−	-		-		1	30.05
	N+	-		-		3	30.44
SPAD	N−	-		-		1	33.00
	N+	-		-		2	40.93
LLE	N−	-		-		2	40.28
	N+	-		-		3	57.53

**Table 4:** List of 64 QTL controlling 12 traits under N− and N+ treatments in population WM-800. Trait abbreviations are indicated in Table 1.

QTL ^a^	Trait	Treatment	DAT ^b^	Chromosome	Start (in bp) ^c^	End (in bp) ^c^	Start (in cM) ^d^	End (in cM) ^d^	−log10 BON_P ^e^	R^2^ [%] ^f^	Effect Patras [%] ^g^	Effect Meister [%] ^g^	Effect Linus [%] ^g^	Effect JB Asano [%] ^g^	Effect Tobak [%] ^g^	Effect Safari [%] ^g^	Effect Bernstein [%] ^g^	Effect Julius [%] ^g^
QSRL3_N-_WM-800_5A	SRL	N−	3	5A	555,179,255	555,936,898	79.55	80.34	4.63	10.51	**18.32**	−7.42	−2.15	**18.32**	−7.42	−2.15	−7.42	−7.42
QSRL3_N+_WM-800_7B	SRL	N+	3	7B	598,478,114	598,478,114	98.30	98.30	4.35	9.80	**15.77**	**15.77**	**−15.77**	**15.77**	**15.77**	**−15.77**	**−15.77**	**15.77**
QSRL7_N+_WM-800_2B	SRL	N+	7	2B	33,550,944	33,940,029	55.44	56.27	1.48	6.26	8.22	8.22	8.22	**−10.08**	8.22	8.22	−0.95	**−10.08**
QSRL7_N+_WM-800_2D	SRL	N+	7	2D	360,697,130	360,697,130	71.50	71.50	3.12	8.54	**10.06**	**−10.06**	**−10.06**	**10.06**	**10.06**	**−10.06**	**−10.06**	**10.06**
QSRL12_N-_WM-800_2D	SRL	N−	12	2D	5,098,920	5,764,428	1.24	1.41	3.03	8.22	**12.01**	**−12.01**	**−12.01**	**12.01**	**12.01**	**−12.01**	**−12.01**	**12.01**
QSRL12_N-_WM-800_5A	SRL	N−	12	5A	688,281,369	691,827,873	124.59	138.18	2.32	9.61	−6.82	0.00	1.11	**19.96**	−4.31	0.45	0.45	−6.82
QSRL12_N+_WM-800_2A	SRL	N+	12	2A	523,988,087	524,855,738	104.13	104.13	3.14	8.93	**−13.26**	**13.26**	**−13.26**	**−13.26**	**13.26**	**−13.26**	**13.26**	**13.26**
QSRL12_N+_WM-800_7D	SRL	N+	12	7D	95,838,332	95,838,332	109.45	109.45	1.45	6.27	**9.02**	**−9.02**	**−9.02**	**9.02**	**−9.02**	**−9.02**	**−9.02**	**9.02**
QLRL3_N+_WM-800_5D	LRL	N+	3	5D	164,652,395	164,652,395	69.13	69.13	2.17	18.69	**−116.13**	**−116.13**	**−116.13**	**−116.13**	**−116.13**	**−116.13**	**116.13**	**−116.13**
QLRL3_N+_WM-800_6B	LRL	N+	3	6B	337,938,852	338,004,900	64.85	64.86	1.74	14.36	**−113.76**	**113.76**	**−113.76**	**−113.76**	**113.76**	**−113.76**	**113.76**	**−113.76**
QLRL7_N-_WM-800_2A	LRL	N−	7	2A	715,301,715	717,582,073	120.18	124.93	1.74	21.03	0.00	−29.27	**213.72**	−29.27	−350.75	−0.71	15.38	−350.75
QLRL12_N+_WM-800_3D	LRL	N+	12	3D	578,414,376	578,675,022	131.22	131.61	1.58	8.87	−18.73	−1.10	−18.73	−18.73	−1.10	**59.69**	−1.10	−18.73
QRSD3_N-_WM-800_3B	RSD	N−	3	3B	492,207,443	492,571,619	68.71	69.03	1.37	9.00	**−25.67**	16.52	16.52	16.52	**−25.67**	**−25.67**	16.52	16.52
QRSD3_N-_WM-800_4A	RSD	N−	3	4A	602,904,647	603,677,217	63.68	65.03	2.48	9.20	3.17	−3.59	−10.49	−8.49	−6.14	**18.71**	1.21	1.21
QRSD3_N+_WM-800_3A	RSD	N+	3	3A	585,022,380	585,022,380	91.92	91.92	1.79	6.99	**13.67**	**13.67**	**13.67**	**−13.67**	**13.67**	**13.67**	**−13.67**	**−13.67**
QRSD7_N-_WM-800_5A	RSD	N−	7	5A	688,281,369	691,827,873	124.59	138.18	5.76	16.36	−10.75	0.00	−1.93	**33.45**	−4.66	2.70	2.70	−10.75
QRSD7_N+_WM-800_6B	RSD	N+	7	6B	440,758,044	440,758,044	66.36	66.36	2.62	7.80	**−16.71**	**−16.71**	**16.71**	**−16.71**	**−16.71**	**−16.71**	**−16.71**	**−16.71**
QRSW3_N-_WM-800_3B	RSW	N−	3	3B	492,207,443	497,090,053	66.78	69.03	3.09	17.74	**−51.85**	**56.23**	**56.23**	**56.23**	**−51.85**	**−51.85**	**56.23**	**56.23**
QRSW3_N-_WM-800_4A	RSW	N−	3	4A	602,904,647	603,677,217	63.68	65.03	1.32	7.50	1.44	−11.54	−16.34	−0.21	−8.51	**27.54**	0.08	0.08
QRSW3_N-_WM-800_6B	RSW	N−	3	6B	25,086,366	26,633,917	25.82	36.69	2.70	8.57	−4.23	**32.65**	−4.23	−4.23	−3.68	−3.68	−15.43	0.00
QRSW3_N+_WM-800_1B	RSW	N+	3	1B	287,707,727	287,708,242	65.50	65.50	4.97	10.56	**23.73**	**23.73**	**−21.34**	**23.73**	**23.73**	**23.73**	−14.69	**−21.34**
QRSW3_N+_WM-800_4A	RSW	N+	3	4A	724,779,492	724,807,687	143.13	143.16	1.36	5.12	**16.06**	**16.06**	−14.19	**16.06**	−11.64	−11.64	**16.06**	**16.06**
QRSW3_N+_WM-800_6D	RSW	N+	3	6D	18,007,821	18,014,962	39.93	39.94	2.50	6.90	−15.35	−15.35	**20.76**	−15.35	−15.35	−5.08	**20.76**	−15.35
QRSW3_N+_WM-800_7A	RSW	N+	3	7A	650,626,928	650,626,928	148.43	148.43	2.31	6.46	**−18.17**	**18.17**	**18.17**	**−18.17**	**−18.17**	**−18.17**	**−18.17**	**18.17**
QRSW7_N-_WM-800_2D	RSW	N−	7	2D	427,955,285	427,956,336	75.37	75.37	1.66	11.96	−6.90	−7.94	−6.90	−6.90	−7.94	**74.89**	−7.94	−6.90
QRSW7_N-_WM-800_5A	RSW	N−	7	5A	688,281,369	691,827,873	124.59	138.18	5.12	14.90	−4.88	0.00	−8.44	**50.05**	−12.64	−0.60	−0.60	−4.88
QRSW7_N+_WM-800_2B	RSW	N+	7	2B	87,474,251	87,918,837	82.92	82.97	2.67	7.98	**18.50**	**−18.91**	**−18.91**	**18.50**	**−18.91**	**−18.91**	**−18.91**	10.55
QRSW7_N+_WM-800_2D	RSW	N+	7	2D	553,894,783	554,126,898	61.65	61.65	1.60	6.65	−5.00	−2.47	**28.14**	−5.00	−2.47	−2.47	−5.00	−5.00
QRSW12_N+_WM-800_6D	RSW	N+	12	6D	302,057,876	302,057,876	83.06	83.06	4.68	12.74	**−31.29**	**31.29**	**31.29**	**−31.29**	**31.29**	**−31.29**	**31.29**	**−31.29**
QCHA3_N-_WM-800_5D	CHA	N−	3	5D	426,640,866	426,640,866	75.00	75.00	1.58	6.26	**−24.74**	**24.74**	**24.74**	**−24.74**	**−24.74**	**−24.74**	**−24.74**	**24.74**
QCHA3_N-_WM-800_6D	CHA	N−	3	6D	62,505,318	62,505,318	74.93	74.93	3.03	8.42	**−31.55**	**−31.55**	**31.55**	**−31.55**	**−31.55**	**31.55**	**−31.55**	**−31.55**
QCHA3_N+_WM-800_1B	CHA	N+	3	1B	280,714,205	280,714,205	64.88	64.88	3.03	8.08	**25.00**	**25.00**	**−25.00**	**25.00**	**25.00**	**25.00**	**−25.00**	**−25.00**
QCHA3_N+_WM-800_2D	CHA	N+	3	2D	553,894,783	554,126,898	61.65	61.65	3.31	8.49	−9.69	−3.47	**44.80**	−9.69	−3.47	−3.47	−9.69	−9.69
QCHA3_N+_WM-800_3B	CHA	N+	3	3B	773,844,953	774,943,447	108.42	109.38	2.47	7.04	**−23.06**	**23.06**	**−23.06**	**−23.06**	**−23.06**	**−23.06**	**23.06**	**−23.06**
QCHA7_N-_WM-800_6B	CHA	N−	7	6B	25,086,366	26,633,917	25.82	36.69	1.82	7.54	−3.91	**35.55**	−3.91	−3.91	−5.28	−5.28	−10.92	0.00
QCHA7_N+_WM-800_7D	CHA	N+	7	7D	203,945	482,251	0.69	1.63	3.47	9.32	−1.92	**43.90**	−1.92	−15.74	−1.92	−15.74	−15.74	−1.92
QCHA12_N-_WM-800_7D	CHA	N−	12	7D	499,886,686	499,886,686	139.28	139.28	2.76	9.65	**−28.98**	**28.98**	**28.98**	**−28.98**	**28.98**	**28.98**	**28.98**	**−28.98**
QCHA12_N+_WM-800_7A	CHA	N+	12	7A	32,531,476	33,246,377	63.32	64.13	2.16	9.31	**34.63**	**34.63**	**−38.79**	**34.63**	**34.63**	−13.88	**−38.79**	**34.63**
QSRA3_N+_WM-800_1D	SRA	N+	3	1D	459,837,376	459,837,376	139.31	139.31	1.33	7.27	**22.09**	**−22.09**	**−22.09**	**−22.09**	**−22.09**	**−22.09**	**−22.09**	**−22.09**
QSRA7_N-_WM-800_5A	SRA	N−	7	5A	688,281,369	691,827,873	124.59	138.18	2.77	11.91	3.11	0.00	−6.85	**30.04**	−9.16	−3.85	−3.85	3.11
QSRA7_N-_WM-800_6B	SRA	N−	7	6B	25,086,366	26,633,917	25.82	36.69	2.81	8.80	−5.25	**24.61**	−5.25	−5.25	−0.16	−0.16	−11.16	0.00
QSRA7_N+_WM-800_1D	SRA	N+	7	1D	455,101,203	455,101,203	134.56	134.56	1.75	6.55	**−13.85**	**−13.85**	**−13.85**	**13.85**	**−13.85**	**13.85**	**−13.85**	**−13.85**
QSRA12_N+_WM-800_3A	SRA	N+	12	3A	743,192,684	744,479,192	177.24	188.38	1.36	9.28	**−21.49**	11.98	**−21.49**	15.08	11.98	−5.77	**−21.49**	9.04
QRRL3_N-_WM-800_7A	RRL	N−	3	7A	652,494,350	653,438,346	148.43	148.43	1.48	8.40	−4.31	5.02	**12.74**	−4.31	−13.19	−13.19	−4.31	**12.74**
QRRL7_N-_WM-800_5A	RRL	N−	7	5A	688,281,369	691,827,873	124.59	138.18	1.32	8.48	−1.72	0.00	3.13	**−16.41**	6.08	2.11	2.11	−1.72
QRRL7_N-_WM-800_6B	RRL	N−	7	6B	33,589,972	33,717,276	40.31	40.90	1.54	6.45	**−9.87**	**−9.87**	8.84	**−9.87**	**−9.87**	3.26	7.52	**−9.87**
QRRL7_N+_WM-800_6A	RRL	N+	7	6A	178,462,401	178,462,401	79.08	79.08	1.56	6.20	**8.15**	**8.15**	**8.15**	**−8.15**	**8.15**	**8.15**	**−8.15**	**−8.15**
QRRR3_N-_WM-800_3B	RRR	N−	3	3B	659,786,600	659,786,600	80.69	80.69	2.38	8.60	**9.59**	**9.59**	**9.59**	**−9.59**	**9.59**	**9.59**	**−9.59**	**−9.59**
QRRR3_N+_WM-800_3D	RRR	N+	3	3D	523,171,752	523,812,634	159.88	160.10	1.84	11.48	0.00	−5.11	−5.11	−5.11	−9.36	**13.07**	0.00	−5.11
QRRR7_N+_WM-800_4B	RRR	N+	7	4B	482,122,893	482,142,092	66.89	66.89	1.37	6.40	**6.85**	**6.85**	**−6.85**	**6.85**	**−6.85**	**6.85**	**−6.85**	**6.85**
QRRR7_N+_WM-800_6D	RRR	N+	7	6D	440,661,063	440,661,063	111.26	111.26	2.58	7.42	**11.25**	**11.25**	**−11.25**	**11.25**	**11.25**	**11.25**	**11.25**	**11.25**
QSDW12_N+_WM-800_4D	SDW	N+	12	4D	18,781,062	18,781,062	69.21	69.21	2.26	6.84	**−9.45**	**9.45**	**−9.45**	**−9.45**	**9.45**	**9.45**	**9.45**	**−9.45**
QR:S12_N-_WM-800_6B	R:S	N−	12	6B	694,119,097	697,021,180	99.14	105.40	1.54	14.92	−6.90	0.00	0.00	−8.50	0.00	0.00	−1.74	−5.43
QR:S12_N+_WM-800_2A	R:S	N+	12	2A	698,825,606	698,827,008	119.39	119.39	3.76	8.90	**−6.90**	**−6.90**	2.33	**−6.90**	**−6.90**	**−6.90**	**10.55**	**−6.90**
QR:S12_N+_WM-800_5B	R:S	N+	12	5B	185,116,588	185,511,485	42.94	42.95	2.08	7.61	−1.48	−1.48	−5.87	**6.94**	−5.87	−1.48	−2.21	**6.94**
QR:S12_N+_WM-800_6D	R:S	N+	12	6D	197,287,391	197,314,683	82.25	82.25	1.47	6.05	−6.54	3.82	3.71	**−6.54**	3.82	3.82	3.82	3.82
QSPAD12_N-_WM-800_4D	SPAD	N−	12	4D	18,781,062	18,781,062	69.21	69.21	2.49	6.82	**4.11**	**−4.11**	**4.11**	**4.11**	**−4.11**	**−4.11**	**−4.11**	**4.11**
QSPAD12_N+_WM-800_4D	SPAD	N+	12	4D	211,039,892	211,039,892	83.84	83.84	3.33	8.28	**4.33**	**−4.33**	**−4.33**	**4.33**	**−4.33**	**4.33**	**−4.33**	**−4.33**
QSPAD12_N+_WM-800_7A	SPAD	N+	12	7A	563,840,926	564,612,431	134.28	134.32	3.92	9.54	2.97	**−4.30**	**−4.30**	2.97	2.12	**−4.30**	2.12	2.97
QLLE12_N-_WM-800_4B	LLE	N−	12	4B	30,861,580	30,861,580	55.96	55.96	6.18	10.13	**10.26**	**10.26**	**10.26**	**10.26**	**−10.21**	**−10.21**	**10.26**	**10.26**
QLLE12_N-_WM-800_4D	LLE	N−	12	4D	25,989,112	25,989,391	69.21	69.21	8.14	13.97	**−11.13**	**10.93**	**−11.13**	**−11.13**	**10.93**	**10.93**	**10.93**	**−11.13**
QLLE12_N+_WM-800_3D	LLE	N+	12	3D	471,079,565	471,079,565	137.67	137.67	2.25	4.83	**10.12**	**10.12**	**10.12**	**10.12**	**−10.12**	**10.12**	**10.12**	**10.12**
QLLE12_N+_WM-800_4B	LLE	N+	12	4B	30,861,580	30,861,580	55.96	55.96	10.39	13.17	**12.22**	**12.22**	**12.22**	**12.22**	**−13.19**	**−13.19**	**12.22**	**12.22**
QLLE12_N+_WM-800_4D	LLE	N+	12	4D	18,781,062	18,781,062	69.21	69.21	10.43	15.75	**−** **12.33**	**12.33**	**−** **12.33**	**−** **12.33**	**12.33**	**12.33**	**12.33**	**−** **12.33**

^a^ Name of the QTL including information on trait, treatment, population and chromosome; ^b^ days after transplanting the germinated seed to the germination paper; ^c^ start and end of the haploblock or singular SNP, based on physical SNP position in bp taken from [61]; ^d^ start and end of the haploblock or singular SNP, based on genetic SNP position in cM taken from [62]; ^e^ the -log10 Bonferroni corrected *p* value of significant (BON_P < 0.05) haplotypes per haploblock or allele per singular SNP; ^f^ proportion of the genotypic variance explained by significant (BON_P < 0.05) haplotypes per haploblock or allele per singular SNP; ^g^ relative QTL allele effect, calculated by dividing the absolute QTL allele effect by the population mean. Effects of significant (BON_P < 0.05) QTL alleles were highlighted with bold numbers. Red color indicates trait-reducing effects.

## Data Availability

The genetic data presented in this study are openly available in Dryad at https://doi.org/10.5061/dryad.zcrjdfnfk [113]. All raw phenotypic data are included in Table S3.

## Supplementary Materials

The following supporting information can be downloaded at: https://www.mdpi.com/article/10.3390/plants11243520/s1, Figure S1: Pearson’s correlation coefficient (r) plot; Figure S2: Pearson’s correlation coefficient (r) plot.png; Table S1: Founder information; Table S2: Nutrient solution for the N treatments; Table S3: Phenotypic data; Table S4: Tag list, abbreviations and description; Table S5: Summary statistics; Table S6: List with all significant MTAs; Table S7: List of all annotated genes in close proximity to the detected QTL.xlsx.

## References

[1] Reynolds M.P., Pask A.J.D., Mullan D.M. (2012). Physiological Breeding I: Interdisciplinary Approaches to Improve Crop Adaptation.

[2] Hawkesford M., Horst W., Kichey T., Lambers H., Schjoerring J., Møller I.S., White P., Marschner H., Marschner P. (2012). Functions of Macronutrients. Marschner’s Mineral Nutrition of Higher Plants.

[3] Clark C.M., Tilman D. (2008). Loss of Plant Species after Chronic Low-Level Nitrogen Deposition to Prairie Grasslands. Nature.

[4] Gough L., Osenberg C.W., Gross K.L., Collins S.L. (2000). Fertilization Effects on Species Density and Primary Productivity in Herbaceous Plant Communities. Oikos.

[5] Suding K.N., Collins S.L., Gough L., Clark C., Cleland E.E., Gross K.L., Milchunas D.G., Pennings S. (2005). Functional- and Abundance-Based Mechanisms Explain Diversity Loss due to N Fertilization. Proc. Natl. Acad. Sci. USA.

[6] Diaz R.J., Rosenberg R. (2008). Spreading Dead Zones and Consequences for Marine Ecosystems. Science.

[7] Guo J.H., Liu X.J., Zhang Y., Shen J.L., Han W.X., Zhang W.F., Christie P., Goulding K.W.T., Vitousek P.M., Zhang F.S. (2010). Significant Acidification in Major Chinese Croplands. Science.

[8] Davidson E.A. (2009). The Contribution of Manure and Fertilizer Nitrogen to Atmospheric Nitrous Oxide since 1860. Nat. Geosci..

[9] Bouwman A.F., Boumans L.J.M., Batjes N.H. (2002). Emissions of N_2_O and NO from Fertilized Fields: Summary of Available Measurement Data. Glob. Biogeochem. Cycles.

[10] Moll R.H., Kamprath E.J., Jackson W.A. (1982). Analysis and Interpretation of Factors Which Contribute to Efficiency of Nitrogen Utilization 1. Agron. J..

[11] Lynch J.P. (2013). Steep, Cheap and Deep: An Ideotype to Optimize Water and N Acquisition by Maize Root Systems. Ann. Bot..

[12] Wang R.F., An D.G., Hu C.S., Li L.H., Zhang Y.M., Jia Y.G., Tong Y.P. (2011). Relationship between Nitrogen Uptake and Use Efficiency of Winter Wheat Grown in the North China Plain. Crop Pasture Sci..

[13] Forde B.G. (2014). Nitrogen Signalling Pathways Shaping Root System Architecture: An Update. Curr. Opin. Plant Biol..

[14] Meister R., Rajani M.S., Ruzicka D., Schachtman D.P. (2014). Challenges of Modifying Root Traits in Crops for Agriculture. Trends Plant Sci..

[15] Barraclough P.B., Weir A.H., Kuhlmann H. (1991). Factors Affecting the Growth and Distribution of Winter Wheat Roots Under Uk Field Conditions. Developments in Agricultural and Managed Forest Ecology.

[16] Sultan S.E. (2003). Phenotypic Plasticity in Plants: A Case Study in Ecological Development. Evol. Dev..

[17] Forde B., Lorenzo H. (2001). The Nutritional Control of Root Development. Plant Soil.

[18] López-Bucio J., Cruz-Ramίrez A., Herrera-Estrella L. (2003). The Role of Nutrient Availability in Regulating Root Architecture. Curr. Opin. Plant Biol..

[19] Siddiqui M.N., Léon J., Naz A.A., Ballvora A. (2021). Genetics and Genomics of Root System Variation in Adaptation to Drought Stress in Cereal Crops. J. Exp. Bot..

[20] Walch-Liu P., Gan Y., Filleur S., Forde B.G.P., Gan Y., Filleur S., Forde B.G. (2005). Nitrogen Signalling and the Regulation of Root Development. Asp. Appl. Biol..

[21] Melino V.J., Fiene G., Enju A., Cai J., Buchner P., Heuer S. (2015). Genetic Diversity for Root Plasticity and Nitrogen Uptake in Wheat Seedlings. Funct. Plant Biol..

[22] Atkinson J.A., Wingen L.U., Griffiths M., Pound M.P., Gaju O., Foulkes M.J., Le Gouis J., Griffiths S., Bennett M.J., King J. (2015). Phenotyping Pipeline Reveals Major Seedling Root Growth QTL in Hexaploid Wheat. J. Exp. Bot..

[23] Kabir M.R., Liu G., Guan P., Wang F., Khan A.A., Ni Z., Yao Y., Hu Z., Xin M., Peng H. (2015). Mapping QTLs Associated with Root Traits Using Two Different Populations in Wheat (*Triticum aestivum* L.). Euphytica.

[24] Pariyar S.R., Nagel K.A., Lentz J., Galinski A., Wilhelm J., Putz A., Adels S., Heinz K., Frohberg C., Watt M. (2021). Variation in Root System Architecture among the Founder Parents of Two 8-Way MAGIC Wheat Populations for Selection in Breeding. Agronomy.

[25] Li P., Chen F., Cai H., Liu J., Pan Q., Liu Z., Gu R., Mi G., Zhang F., Yuan L. (2015). A Genetic Relationship between Nitrogen Use Efficiency and Seedling Root Traits in Maize as Revealed by QTL Analysis. J. Exp. Bot..

[26] Dai X., Xiao L., Jia D., Kong H., Wang Y., Li C., Zhang Y., He M. (2014). Increased Plant Density of Winter Wheat Can Enhance Nitrogen—Uptake from Deep Soil. Plant Soil.

[27] Liu H., Fiorani F., Jäck O., Colombi T., Nagel K.A., Weih M. (2021). Shoot and Root Traits Underlying Genotypic Variation in Early Vigor and Nutrient Accumulation in Spring Wheat Grown in High-Latitude Light Conditions. Plants.

[28] Gifford M.L., Dean A., Gutierrez R.A., Coruzzi G.M., Birnbaum K.D. (2008). Cell-Specific Nitrogen Responses Mediate Developmental Plasticity. Proc. Natl. Acad. Sci. USA.

[29] Ho C.-H., Lin S.-H., Hu H.-C., Tsay Y.-F. (2009). CHL1 Functions as a Nitrate Sensor in Plants. Cell.

[30] Asim M., Ullah Z., Xu F., An L., Aluko O.O., Wang Q., Liu H. (2020). Nitrate Signaling, Functions, and Regulation of Root System Architecture: Insights from Arabidopsis Thaliana. Genes.

[31] Benková E., Hejátko J. (2009). Hormone Interactions at the Root Apical Meristem. Plant Mol. Biol..

[32] Bishopp A., Ursache R., Helariutta Y. (2012). Plant Development: How Long Is a Root?. Curr. Biol..

[33] Ioio R.D., Nakamura K., Moubayidin L., Perilli S., Taniguchi M., Morita M.T., Aoyama T., Costantino P., Sabatini S. (2008). A Genetic Framework for the Control of Cell Division and Differentiation in the Root Meristem. Science.

[34] Maccaferri M., El-Feki W., Nazemi G., Salvi S., Canè M.A., Colalongo M.C., Stefanelli S., Tuberosa R. (2016). Prioritizing Quantitative Trait Loci for Root System Architecture in Tetraploid Wheat. J. Exp. Bot..

[35] Canè M.A., Maccaferri M., Nazemi G., Salvi S., Francia R., Colalongo C., Tuberosa R. (2014). Association Mapping for Root Architectural Traits in Durum Wheat Seedlings as Related to Agronomic Performance. Mol. Breed. New Strateg. Plant Improv..

[36] Guo Y., Kong F., Xu Y., Zhao Y., Liang X., Wang Y., An D., Li S. (2012). QTL Mapping for Seedling Traits in Wheat Grown under Varying Concentrations of N, P and K Nutrients. Theor. Appl. Genet..

[37] Fan X., Zhang W., Zhang N., Chen M., Zheng S., Zhao C., Han J., Liu J., Zhang X., Song L. (2018). Identification of QTL Regions for Seedling Root Traits and Their Effect on Nitrogen Use Efficiency in Wheat (*Triticum aestivum* L.). Theor. Appl. Genet..

[38] Xie Q., Fernando K.M.C., Mayes S., Sparkes D.L. (2017). Identifying Seedling Root Architectural Traits Associated with Yield and Yield Components in Wheat. Ann. Bot..

[39] Huang B.E., George A.W., Forrest K.L., Kilian A., Hayden M.J., Morell M.K., Cavanagh C.R. (2012). A Multiparent Advanced Generation Inter-Cross Population for Genetic Analysis in Wheat: Mapping a Wheat MAGIC Population. Plant Biotechnol. J..

[40] Mackay I.J., Bansept-Basler P., Barber T., Bentley A.R., Cockram J., Gosman N., Greenland A.J., Horsnell R., Howells R., O’Sullivan D.M. (2014). An Eight-Parent Multiparent Advanced Generation Inter-Cross Population for Winter-Sown Wheat: Creation, Properties, and Validation. G3 Genes Genomes Genet..

[41] Stadlmeier M., Hartl L., Mohler V. (2018). Usefulness of a Multiparent Advanced Generation Intercross Population with a Greatly Reduced Mating Design for Genetic Studies in Winter Wheat. Front. Plant Sci..

[42] Qian L., Hickey L.T., Stahl A., Werner C.R., Hayes B., Snowdon R.J., Voss-Fels K.P. (2017). Exploring and Harnessing Haplotype Diversity to Improve Yield Stability in Crops. Front. Plant Sci..

[43] Sehgal D., Mondal S., Crespo-Herrera L., Velu G., Juliana P., Huerta-Espino J., Shrestha S., Poland J., Singh R., Dreisigacker S. (2020). Haplotype-Based, Genome-Wide Association Study Reveals Stable Genomic Regions for Grain Yield in CIMMYT Spring Bread Wheat. Front. Genet..

[44] Chen S., Liu F., Wu W., Jiang Y., Zhan K. (2021). A SNP-Based GWAS and Functional Haplotype-Based GWAS of Flag Leaf-Related Traits and Their Influence on the Yield of Bread Wheat (*Triticum aestivum* L.). Theor. Appl. Genet..

[45] Ogawa D., Nonoue Y., Tsunematsu H., Kanno N., Yamamoto T., Yonemaru J. (2018). Discovery of QTL Alleles for Grain Shape in the Japan-MAGIC Rice Population Using Haplotype Information. G3 Genes Genomes Genet..

[46] Lisker A., Maurer A., Schmutzer T., Kazman E., Cöster H., Holzapfel J., Ebmeyer E., Alqudah A.M., Sannemann W., Pillen K. (2022). A Haplotype-Based GWAS Identified Trait-Improving QTL Alleles Controlling Grain Yield under Nitrogen Fertilization Treatment.

[47] Sannemann W., Lisker A., Maurer A., Léon J., Kazman E., Cöster H., Holzapfel J., Kempf H., Korzun V., Ebmeyer E. (2018). Adaptive Selection of Founder Segments and Epistatic Control of Plant Height in the MAGIC Winter Wheat Population WM-800. BMC Genom..

[48] Schmidt L., Jacobs J., Schmutzer T., Alqudah A.M., Sannemann W., Pillen K., Maurer A. (2022). Identifying Genomic Regions Determining Nitrogen Uptake Efficiency of Shoot and Root Traits in a Multiparent Advanced Generation Intercross (MAGIC) Winter Wheat Population in a High-Throughput Phenotyping Facility.

[49] Petrarulo M., Marone D., Ferragonio P., Cattivelli L., Rubiales D., de Vita P., Mastrangelo A.M. (2015). Genetic Analysis of Root Morphological Traits in Wheat. Mol. Genet. Genom..

[50] Péret B., de Rybel B., Casimiro I., Benková E., Swarup R., Laplaze L., Beeckman T., Bennett M.J. (2009). Arabidopsis Lateral Root Development: An Emerging Story. Trends Plant Sci..

[51] Osmont K.S., Sibout R., Hardtke C.S. (2007). Hidden Branches: Developments in Root System Architecture. Annu. Rev. Plant Biol..

[52] Wang X., Bian Y., Cheng K., Zou H., Sun S.S.-M., He J.-X. (2012). A Comprehensive Differential Proteomic Study of Nitrate Deprivation in Arabidopsis Reveals Complex Regulatory Networks of Plant Nitrogen Responses. J. Proteome Res..

[53] Guo T., Xuan H., Yang Y., Wang L., Wei L., Wang Y., Kang G. (2014). Transcription Analysis of Genes Encoding the Wheat Root Transporter NRT1 and NRT2 Families During Nitrogen Starvation. J. Plant Growth Regul..

[54] Krouk G., Lacombe B., Bielach A., Perrine-Walker F., Malinska K., Mounier E., Hoyerova K., Tillard P., Leon S., Ljung K. (2010). Nitrate-Regulated Auxin Transport by NRT1.1 Defines a Mechanism for Nutrient Sensing in Plants. Dev. Cell.

[55] Naulin P.A., Armijo G.I., Vega A.S., Tamayo K.P., Gras D.E., de la Cruz J., Gutiérrez R.A. (2020). Nitrate Induction of Primary Root Growth Requires Cytokinin Signaling in Arabidopsis Thaliana. Plant Cell Physiol..

[56] Drew M.C. (1975). Comparison of the Effects of a localised Supply of Phosphate, Nitrate, Ammonium and Potassium on the Growth of the Seminal Root System, and the Shoot, in Barley. New Phytol..

[57] Schneider H.M., Lynch J.P. (2020). Should Root Plasticity Be a Crop Breeding Target?. Front. Plant Sci..

[58] Gruber B.D., Giehl R.F.H., Friedel S., von Wirén N. (2013). Plasticity of the Arabidopsis Root System under Nutrient Deficiencies. Plant Physiol..

[59] Brancourt-Hulmel M., Heumez E., Pluchard P., Beghin D., Depatureaux C., Giraud A., Gouis J. (2005). Indirect versus Direct Selection of Winter Wheat for Low-Input or High-Input Levels. Crop Sci..

[60] Presterl T., Seitz G., Landbeck M., Thiemt E.M., Schmidt W., Geiger H.H. (2003). Improving Nitrogen–Use Efficiency in European Maize. Crop Sci..

[61] Alaux M., Rogers J., Letellier T., Flores R., Alfama F., Pommier C., Mohellibi N., Durand S., Kimmel E., Michotey C. (2018). Linking the International Wheat Genome Sequencing Consortium Bread Wheat Reference Genome Sequence to Wheat Genetic and Phenomic Data. Genome Biol..

[62] Wang S., Wong D., Forrest K., Allen A., Chao S., Huang B.E., Maccaferri M., Salvi S., Milner S.G., Cattivelli L. (2014). Characterization of Polyploid Wheat Genomic Diversity Using a High-Density 90,000 Single Nucleotide Polymorphism Array. Plant Biotechnol. J..

[63] Bai C., Liang Y., Hawkesford M.J. (2013). Identification of QTLs Associated with Seedling Root Traits and Their Correlation with Plant Height in Wheat. J. Exp. Bot..

[64] Wojciechowski T., Gooding M.J., Ramsay L., Gregory P.J. (2009). The Effects of Dwarfing Genes on Seedling Root Growth of Wheat. J. Exp. Bot..

[65] Li P., Chen J., Wu P., Zhang J., Chu C., See D., Brown-Guedira G., Zemetra R., Souza E. (2011). Quantitative Trait Loci Analysis for the Effect of Rht-B1 Dwarfing Gene on Coleoptile Length and Seedling Root Length and Number of Bread Wheat. Crop Sci..

[66] Peng J., Richards D.E., Hartley N.M., Murphy G.P., Devos K.M., Flintham J.E., Beales J., Fish L.J., Worland A.J., Pelica F. (1999). “Green Revolution” Genes Encode Mutant Gibberellin Response Modulators. Nature.

[67] Rao V., Petla B.P., Verma P., Salvi P., Kamble N.U., Ghosh S., Kaur H., Saxena S.C., Majee M. (2018). Arabidopsis SKP1-like Protein13 (ASK13) Positively Regulates Seed Germination and Seedling Growth under Abiotic Stress. J. Exp. Bot..

[68] Li C., Liu Z., Zhang Q., Wang R., Xiao L., Ma H., Chong K., Xu Y. (2012). SKP1 Is Involved in Abscisic Acid Signalling to Regulate Seed Germination, Stomatal Opening and Root Growth in Arabidopsis Thaliana: SKP1 Is Involved in ABA Signalling. Plant Cell Environ..

[69] Li C., Liang Y., Chen C., Li J., Xu Y., Xu Z., Ma H., Chong K. (2006). Cloning and Expression Analysis of TSK1, a Wheat SKP1 Homologue, and Functional Comparison with Arabidopsis ASK1 in Male Meiosis and Auxin Signalling. Funct. Plant Biol..

[70] Borrill P., Ramirez-Gonzalez R., Uauy C. (2016). ExpVIP: A Customizable RNA-Seq Data Analysis and Visualization Platform. Plant Physiol..

[71] Ramírez-González R.H., Borrill P., Lang D., Harrington S.A., Brinton J., Venturini L., Davey M., Jacobs J., van Ex F., Pasha A. (2018). The Transcriptional Landscape of Polyploid Wheat. Science.

[72] Munnik T., Testerink C. (2009). Plant Phospholipid Signaling: “In a Nutshell”. J. Lipid Res..

[73] Hetherington A.M., Brownlee C. (2004). The generation of Ca^2+^ signals in plants. Annu. Rev. Plant Biol..

[74] De Silva K., Laska B., Brown C., Sederoff H.W., Khodakovskaya M. (2011). Arabidopsis Thaliana Calcium-Dependent Lipid-Binding Protein (AtCLB): A Novel Repressor of Abiotic Stress Response. J. Exp. Bot..

[75] Li X., Zhang Q., Yang X., Han J., Zhu Z. (2019). OsANN3, a Calcium-Dependent Lipid Binding Annexin Is a Positive Regulator of ABA-Dependent Stress Tolerance in Rice. Plant Sci..

[76] Li M., Wang T., Zhang H., Liu S., Li W., Abou Elwafa S.F., Tian H. (2022). TaNRT2.1-6B Is a Dual-Affinity Nitrate Transporter Contributing to Nitrogen Uptake in Bread Wheat under Both Nitrogen Deficiency and Sufficiency. Crop J..

[77] Kaur J., Sebastian J., Siddiqi I. (2006). The *Arabidopsis*-*Mei2*-like Genes Play a Role in Meiosis and Vegetative Growth in *Arabidopsis*. Plant Cell.

[78] Terasaka K., Blakeslee J.J., Titapiwatanakun B., Peer W.A., Bandyopadhyay A., Makam S.N., Lee O.R., Richards E.L., Murphy A.S., Sato F. (2005). PGP4, an ATP Binding Cassette P-Glycoprotein, Catalyzes Auxin Transport in *Arabidopsis thaliana* Roots. Plant Cell.

[79] Wu G., Lewis D.R., Spalding E.P. (2007). Mutations in *Arabidopsis* Multidrug Resistance-like ABC Transporters Separate the Roles of Acropetal and Basipetal Auxin Transport in Lateral Root Development. Plant Cell.

[80] Noh B., Murphy A.S., Spalding E.P. (2001). Multidrug Resistance-like Genes of Arabidopsis Required for Auxin Transport and Auxin-Mediated Development. Plant Cell.

[81] Geisler M., Blakeslee J.J., Bouchard R., Lee O.R., Vincenzetti V., Bandyopadhyay A., Titapiwatanakun B., Peer W.A., Bailly A., Richards E.L. (2005). Cellular Efflux of Auxin Catalyzed by the Arabidopsis MDR/PGP Transporter AtPGP1: Auxin Efflux Catalyzed by AtPGP1. Plant J..

[82] Woo H.-H., Orbach M.J., Hirsch A.M., Hawes M.C. (1999). Meristem-Localized Inducible Expression of a UDP-Glycosyltransferase Gene Is Essential for Growth and Development in Pea and Alfalfa. Plant Cell.

[83] Jacobs M., Rubery P.H. (1988). Naturally occurring auxin transport regulators. Science.

[84] McQueen-Mason S., Cosgrove D.J. (1994). Disruption of Hydrogen Bonding between Plant Cell Wall Polymers by Proteins That Induce Wall Extension. Proc. Natl. Acad. Sci. USA.

[85] Cho H.-T., Cosgrove D.J. (2002). Regulation of Root Hair Initiation and Expansin Gene Expression in Arabidopsis. Plant Cell.

[86] Choi D., Lee Y., Cho H.-T., Kende H. (2003). Regulation of Expansin Gene Expression Affects Growth and Development in Transgenic Rice Plants. Plant Cell.

[87] Lee D.-K., Ahn J.H., Song S.-K., Choi Y.D., Lee J.S. (2003). Expression of an Expansin Gene Is Correlated with Root Elongation in Soybean. Plant Physiol..

[88] Ramakrishna P., Ruiz Duarte P., Rance G.A., Schubert M., Vordermaier V., Vu L.D., Murphy E., Vilches Barro A., Swarup K., Moirangthem K. (2019). EXPANSIN A1-Mediated Radial Swelling of Pericycle Cells Positions Anticlinal Cell Divisions during Lateral Root Initiation. Proc. Natl. Acad. Sci. USA.

[89] Li Z., Liu D., Xia Y., Li Z., Jing D., Du J., Niu N., Ma S., Wang J., Song Y. (2020). Identification of the WUSCHEL-Related Homeobox (WOX) Gene Family, and Interaction and Functional Analysis of TaWOX9 and TaWUS in Wheat. Int. J. Mol. Sci..

[90] Zhao Y., Hu Y., Dai M., Huang L., Zhou D.-X. (2009). The WUSCHEL-Related Homeobox Gene *WOX11* Is Required to Activate Shoot-Borne Crown Root Development in Rice. Plant Cell.

[91] Zhu Y., Hu C., Gou X. (2020). Receptor-like Protein Kinase-Mediated Signaling in Controlling Root Meristem Homeostasis. aBIOTECH.

[92] Vaid N., Macovei A., Tuteja N. (2013). Knights in Action: Lectin Receptor-like Kinases in Plant Development and Stress Responses. Mol. Plant.

[93] De Smet I., Signora L., Beeckman T., Inzé D., Foyer C.H., Zhang H. (2003). An Abscisic Acid-Sensitive Checkpoint in Lateral Root Development of *Arabidopsis*. Plant J..

[94] Fujii H., Verslues P.E., Zhu J.-K. (2007). Identification of Two Protein Kinases Required for Abscisic Acid Regulation of Seed Germination, Root Growth, and Gene Expression in *Arabidopsis*. Plant Cell.

[95] Voss-Fels K.P., Robinson H., Mudge S.R., Richard C., Newman S., Wittkop B., Stahl A., Friedt W., Frisch M., Gabur I. (2018). VERNALIZATION1 Modulates Root System Architecture in Wheat and Barley. Mol. Plant.

[96] Kirschner G.K., Rosignoli S., Guo L., Vardanega I., Imani J., Altmüller J., Milner S.G., Balzano R., Nagel K.A., Pflugfelder D. (2021). *ENHANCED GRAVITROPISM 2* Encodes a STERILE ALPHA MOTIF-Containing Protein That Controls Root Growth Angle in Barley and Wheat. Proc. Natl. Acad. Sci. USA.

[97] Beyer S., Daba S., Tyagi P., Bockelman H., Brown-Guedira G., Mohammadi M. (2019). Loci and Candidate Genes Controlling Root Traits in Wheat Seedlings—A Wheat Root GWAS. Funct. Integr. Genom..

[98] Alemu A., Feyissa T., Maccaferri M., Sciara G., Tuberosa R., Ammar K., Badebo A., Acevedo M., Letta T., Abeyo B. (2021). Genome-Wide Association Analysis Unveils Novel QTLs for Seminal Root System Architecture Traits in Ethiopian Durum Wheat. BMC Genom..

[99] Higgins C.F., Payne J.W., Boulter D., Parthier B. (1982). Plant Peptides. Nucleic Acids and Proteins in Plants I.

[100] Zentella R., Sui N., Barnhill B., Hsieh W.-P., Hu J., Shabanowitz J., Boyce M., Olszewski N.E., Zhou P., Hunt D.F. (2017). The Arabidopsis O-Fucosyltransferase SPINDLY Activates Nuclear Growth Repressor DELLA. Nat. Chem. Biol..

[101] Nelson S.K., Steber C.M., Hedden P., Thomas S.G. (2016). Gibberellin Hormone Signal Perception: Down-Regulating DELLA Repressors of Plant Growth and Development. Annual Plant Reviews, the Gibberellins.

[102] Cavanagh C., Morell M., Mackay I., Powell W. (2008). From Mutations to MAGIC: Resources for Gene Discovery, Validation and Delivery in Crop Plants. Curr. Opin. Plant Biol..

[103] Gioia T., Galinski A., Lenz H., Müller C., Lentz J., Heinz K., Briese C., Putz A., Fiorani F., Watt M. (2016). GrowScreen-PaGe, a Non-Invasive, High-Throughput Phenotyping System Based on Germination Paper to Quantify Crop Phenotypic Diversity and Plasticity of Root Traits under Varying Nutrient Supply. Funct. Plant Biol..

[104] RStudio Team (2020). RStudio: Integrated Development for R.

[105] Lenth R. (2022). Emmeans: Estimated Marginal Means, Aka Least-Squares Means. https://CRAN.R-project.org/package=emmeans.

[106] Chung Y., Rabe-Hesketh S., Dorie V., Gelman A., Liu J. (2013). A Nondegenerate Penalized Likelihood Estimator for Variance Parameters in Multilevel Models. Psychometrika.

[107] Voss-Fels K.P., Stahl A., Wittkop B., Lichthardt C., Nagler S., Rose T., Chen T.-W., Zetzsche H., Seddig S., Majid Baig M. (2019). Breeding Improves Wheat Productivity under Contrasting Agrochemical Input Levels. Nat. Plants.

[108] Revelle W. (2022). Psych: Procedures for Psychological, Psychometric, and Personality Research.

[109] Miyagawa T., Nishida N., Ohashi J., Kimura R., Fujimoto A., Kawashima M., Koike A., Sasaki T., Tanii H., Otowa T. (2008). Appropriate Data Cleaning Methods for Genome-Wide Association Study. J. Hum. Genet..

[110] Maurer A., Draba V., Jiang Y., Schnaithmann F., Sharma R., Schumann E., Kilian B., Reif J.C., Pillen K. (2015). Modelling the Genetic Architecture of Flowering Time Control in Barley through Nested Association Mapping. BMC Genom..

[111] Rutkoski J.E., Poland J., Jannink J.-L., Sorrells M.E. (2013). Imputation of Unordered Markers and the Impact on Genomic Selection Accuracy. G3 Genes Genomes Genet..

[112] Barrett J.C., Fry B., Maller J., Daly M.J. (2005). Haploview: Analysis and Visualization of LD and Haplotype Maps. Bioinformatics.

[113] Pillen K., Sannemann W., Lisker A., Maurer A., Schmutzer T., Alqudah A.M. Determining Haploblocks and Haplotypes in the MAGIC Winter Wheat Population WM-800 Based on the Wheat 15k Infinium and the 135k Affymetrix SNP Arrays. Dryad.

[114] Bergelson J., Roux F. (2010). Towards Identifying Genes Underlying Ecologically Relevant Traits in *Arabidopsis thaliana*. Nat. Rev. Genet..

[115] Tuinstra M.R., Ejeta G., Goldsbrough P.B. (1997). Heterogeneous Inbred Family (HIF) Analysis: A Method for Developing Near-Isogenic Lines That Differ at Quantitative Trait Loci. Theor. Appl. Genet..

