# Supplementary material for: Unraveling Genomic Regions Controlling Root Traits as a Function of Nitrogen Availability in the MAGIC Wheat Population WM-800
Source: Plants (Basel). 2022 Dec 14;11(24):3520. doi: 10.3390/plants11243520 (PMC9785272; doi:10.3390/plants11243520)
Supplement: Supplementary file 1 [file plants-11-03520-s001.zip › Figure S1 Box-Whisker-Plots comparing phenotypic varaition between WM-800 lines, founders and check varieties.pdf]

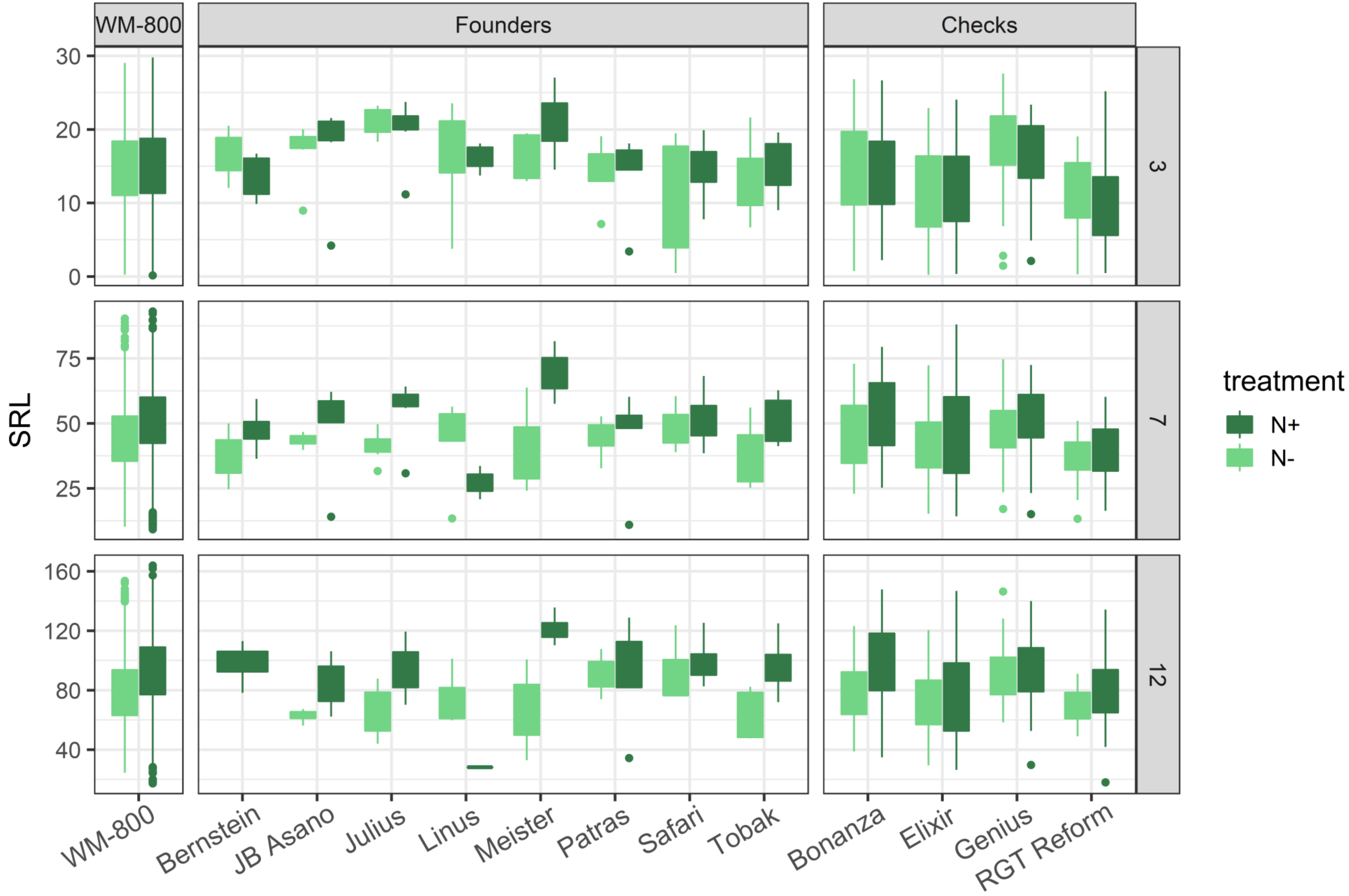

Figure S1 Box-Whisker-Plots comparing phenotypic varaition between WM-800 lines, founders and check varieties

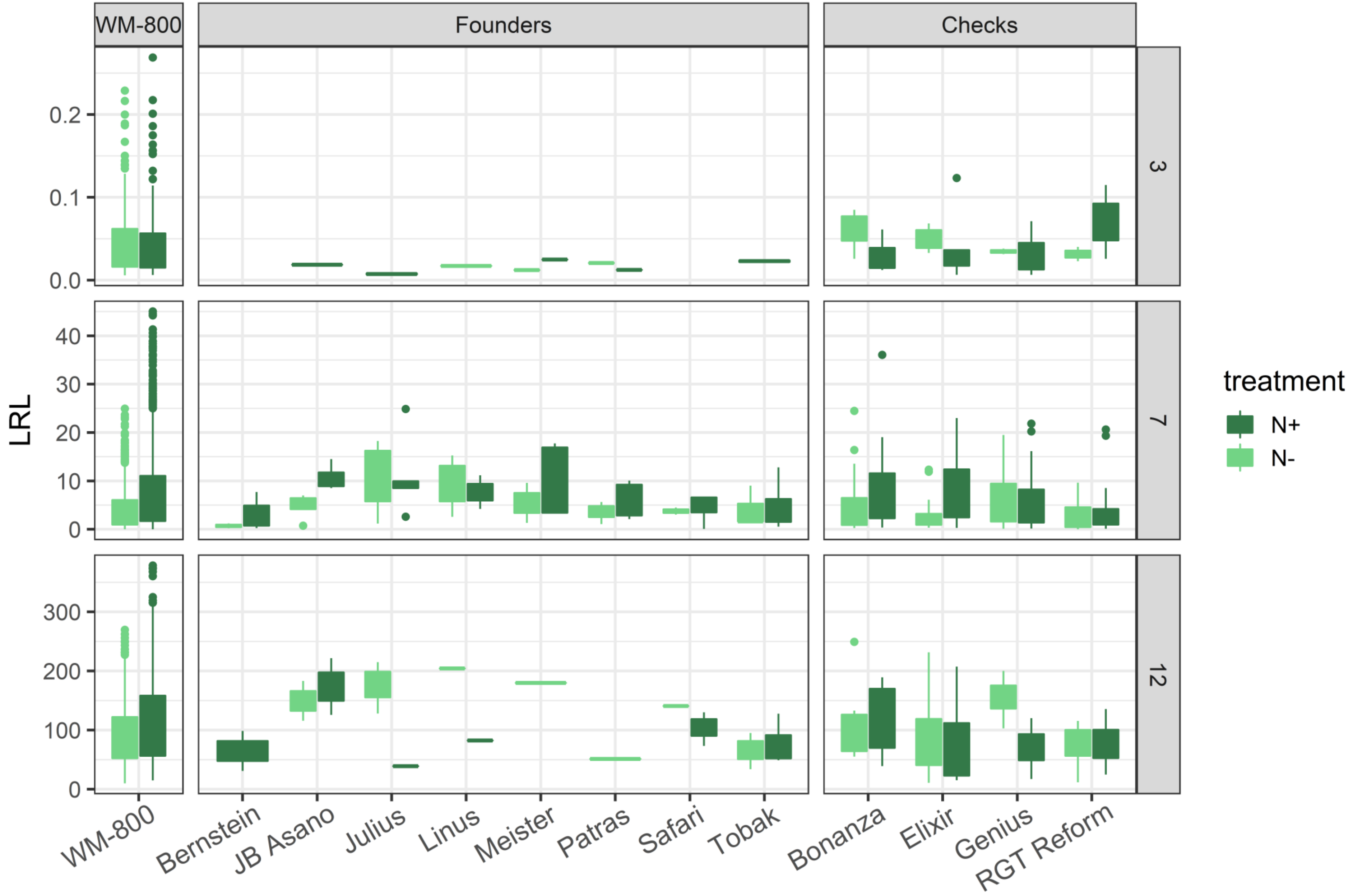

Figure S1 Box-Whisker-Plots comparing phenotypic varaition between WM-800 lines, founders and check varieties

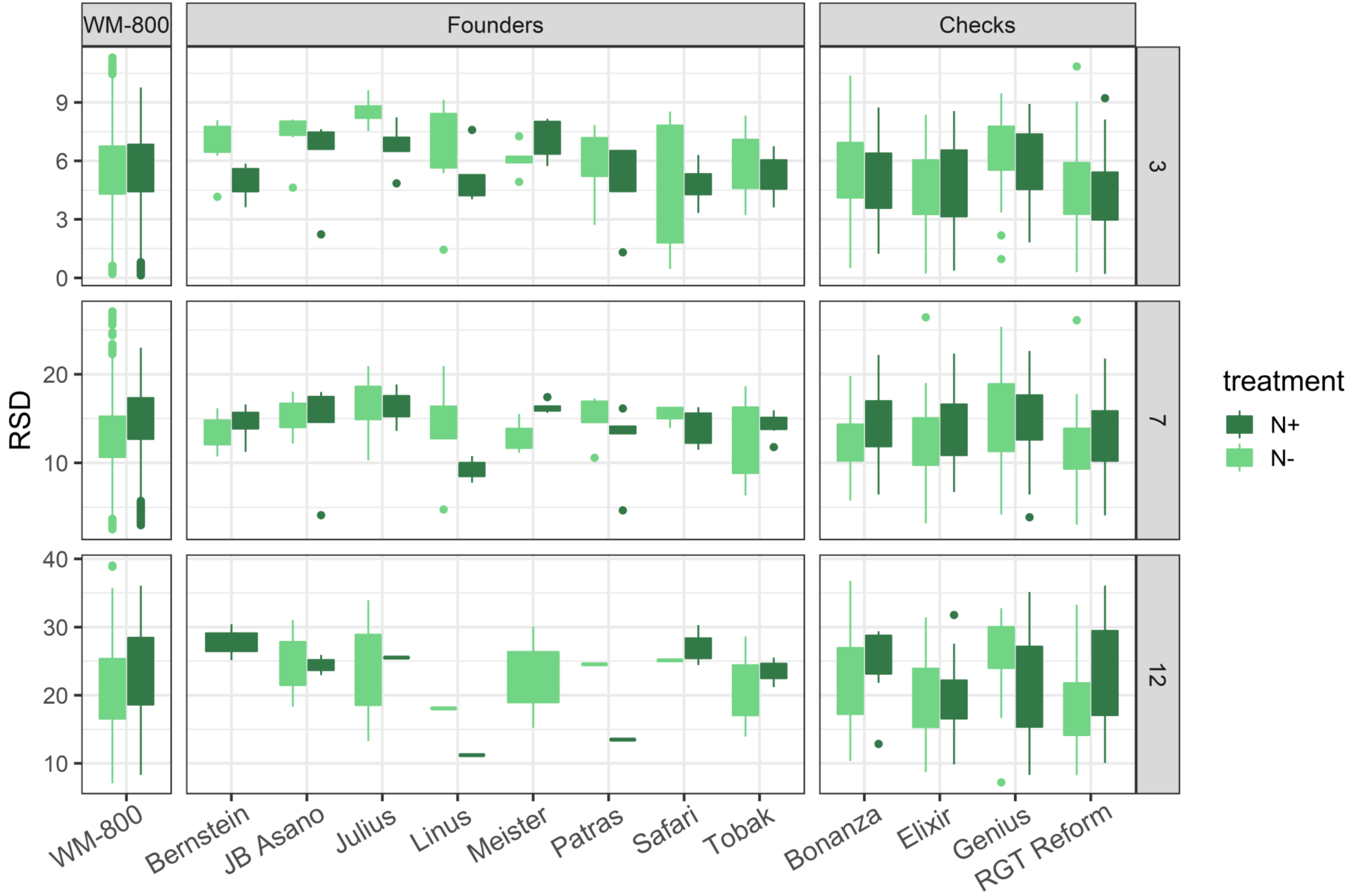

Figure S1 Box-Whisker-Plots comparing phenotypic varaition between WM-800 lines, founders and check varieties

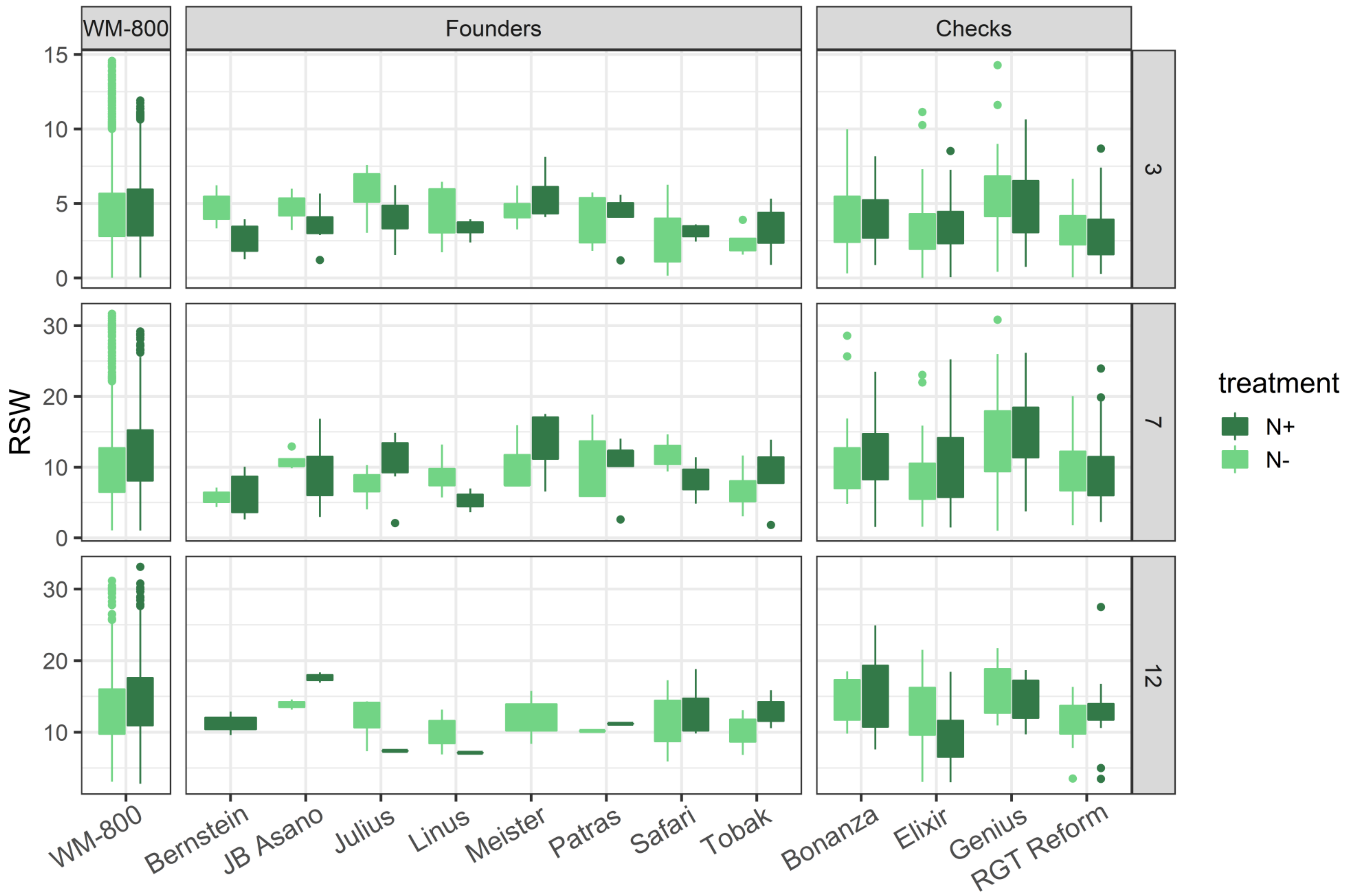

Figure S1 Box-Whisker-Plots comparing phenotypic varaition between WM-800 lines, founders and check varieties

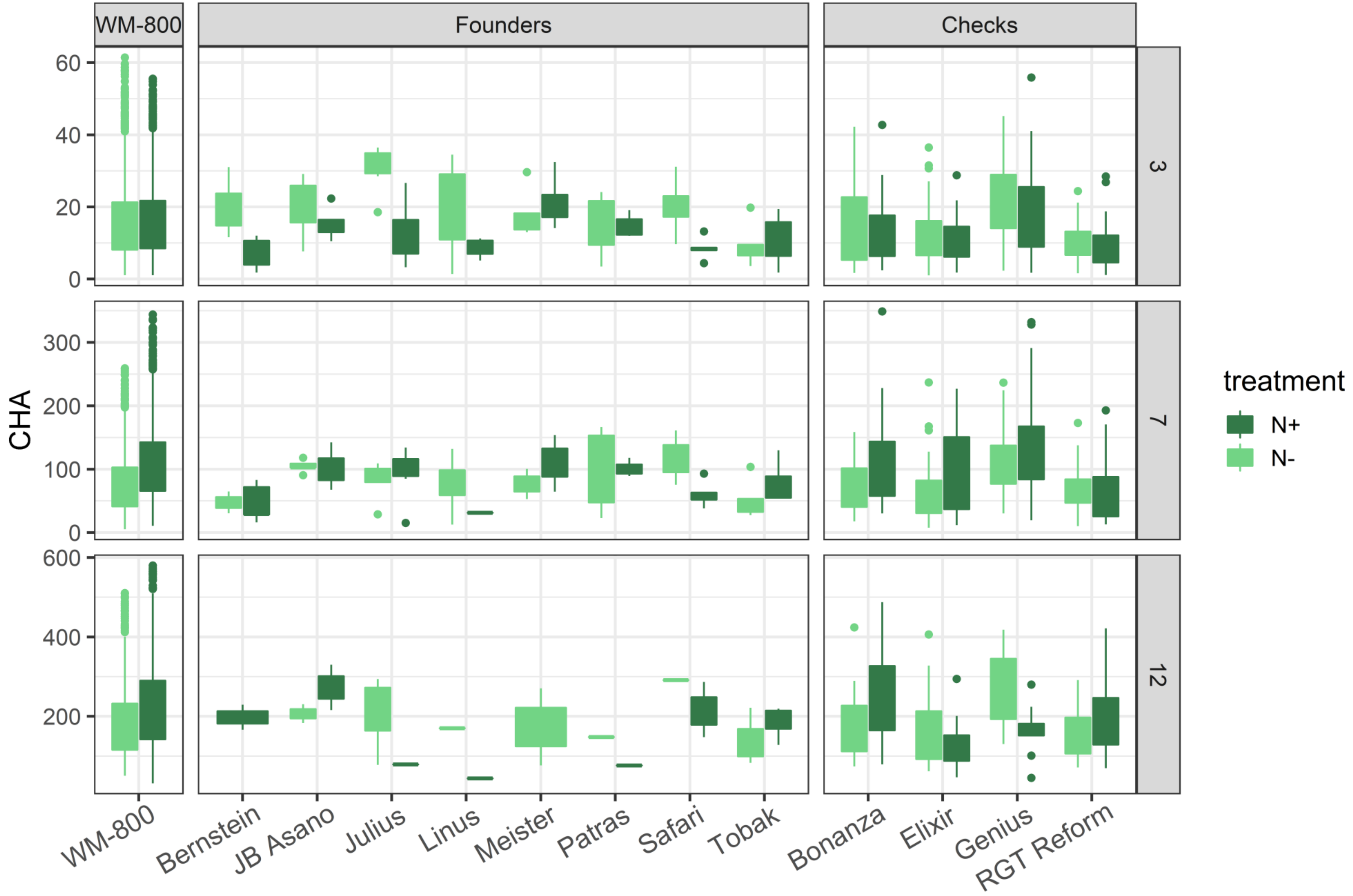

Figure S1 Box-Whisker-Plots comparing phenotypic varaition between WM-800 lines, founders and check varieties

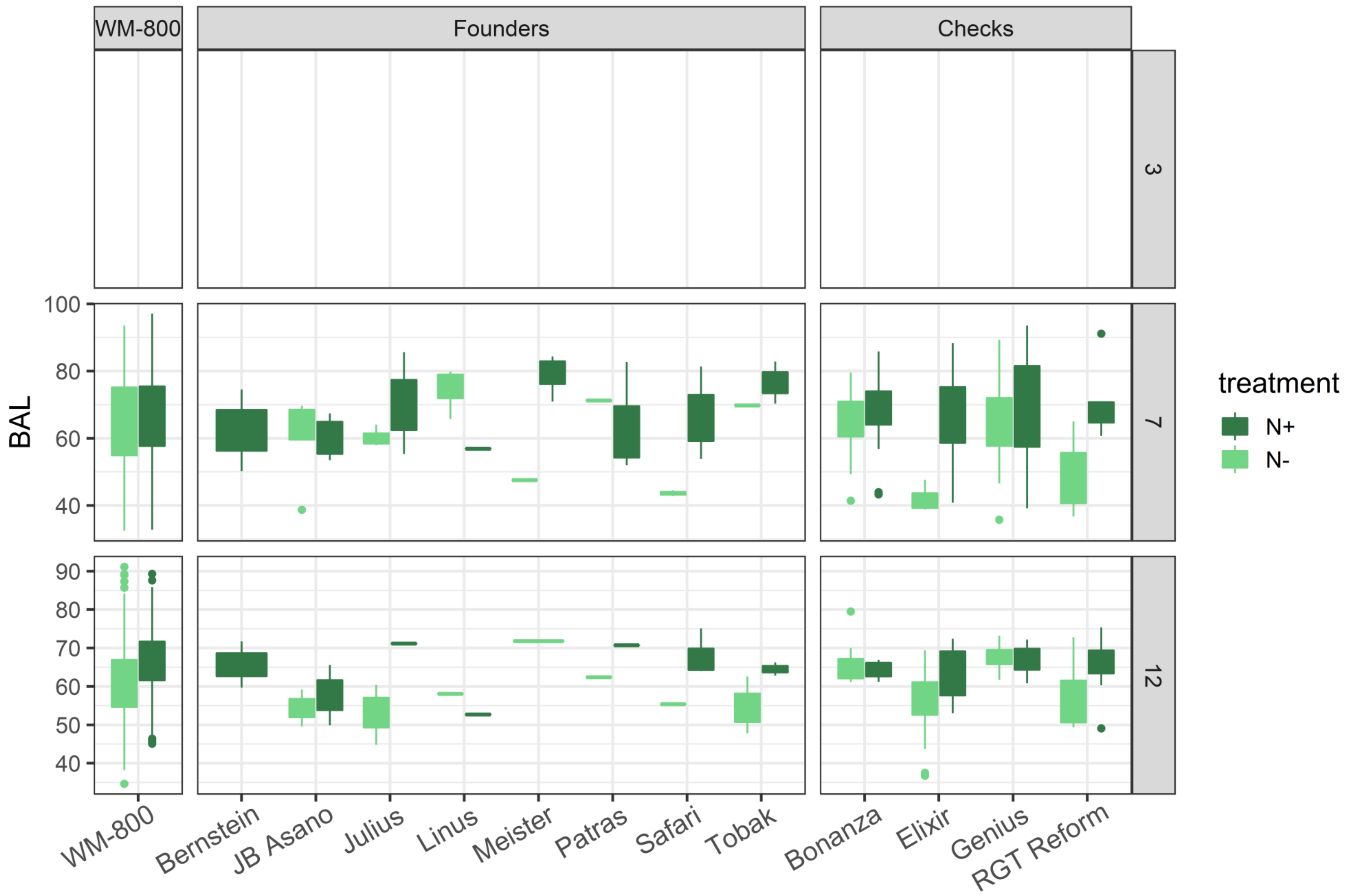

Figure S1 Box-Whisker-Plots comparing phenotypic varaition between WM-800 lines, founders and check varieties

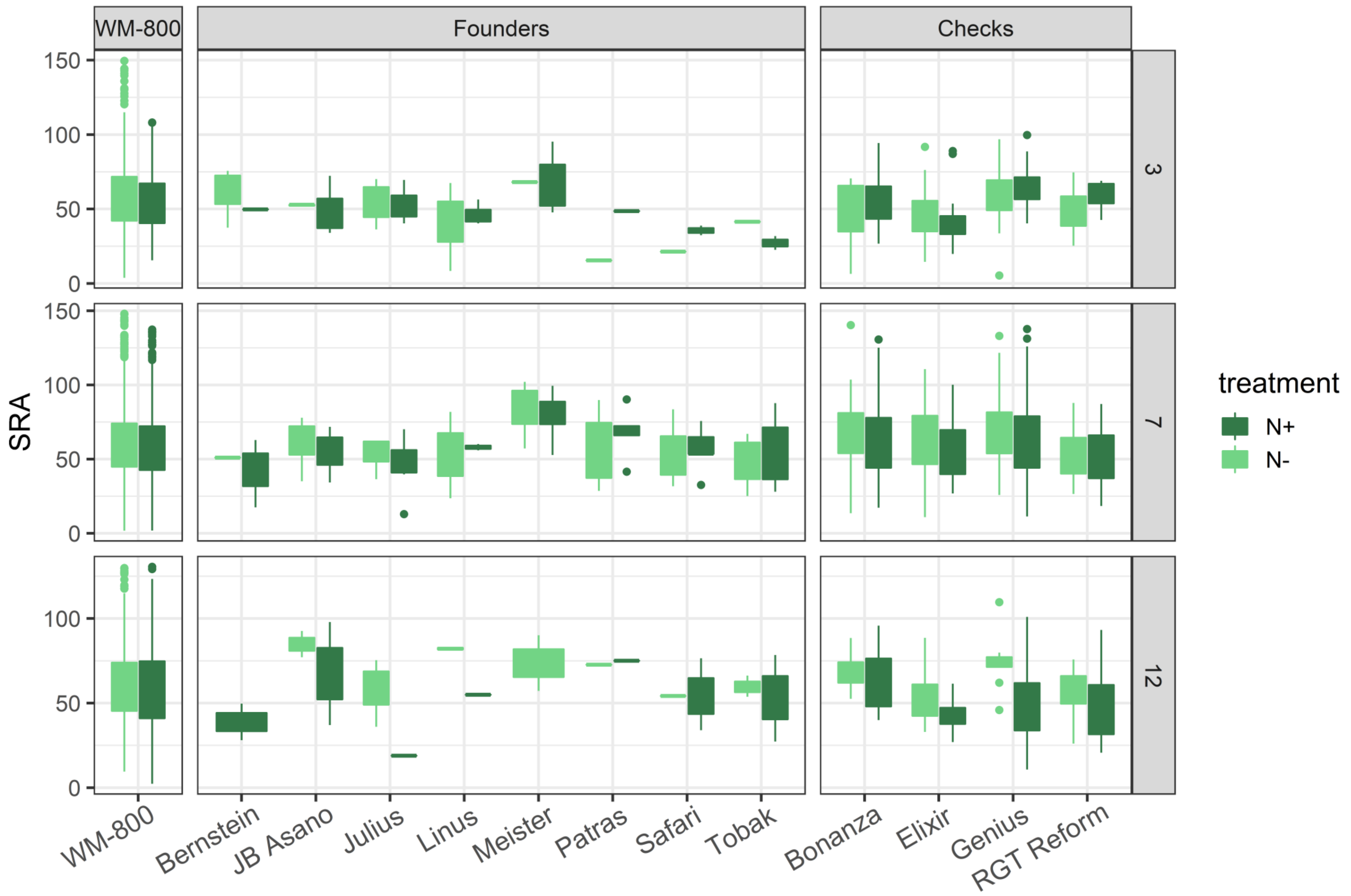

Figure S1 Box-Whisker-Plots comparing phenotypic varaition between WM-800 lines, founders and check varieties

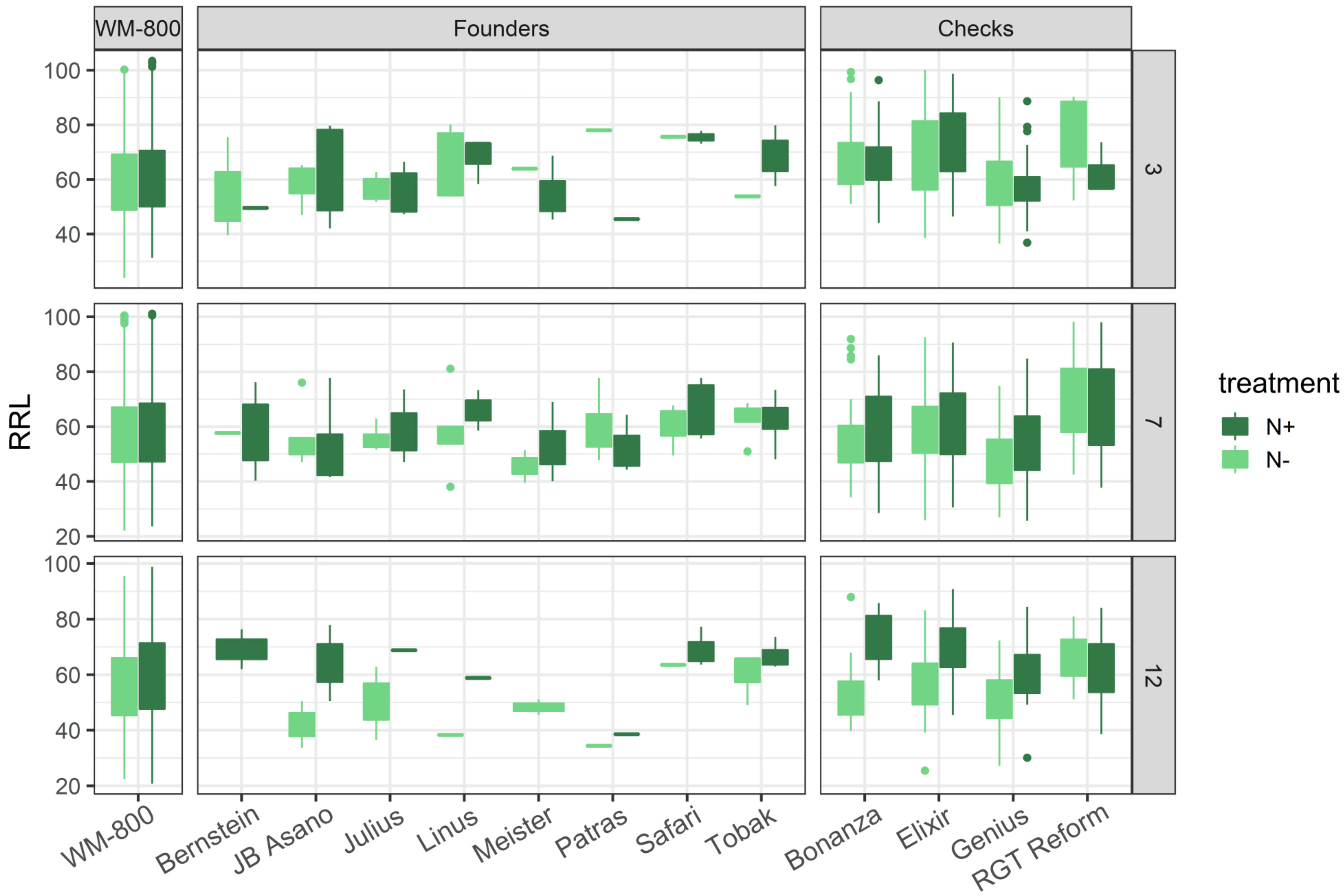

Figure S1 Box-Whisker-Plots comparing phenotypic varaition
between WM-800 lines, founders and check varieties

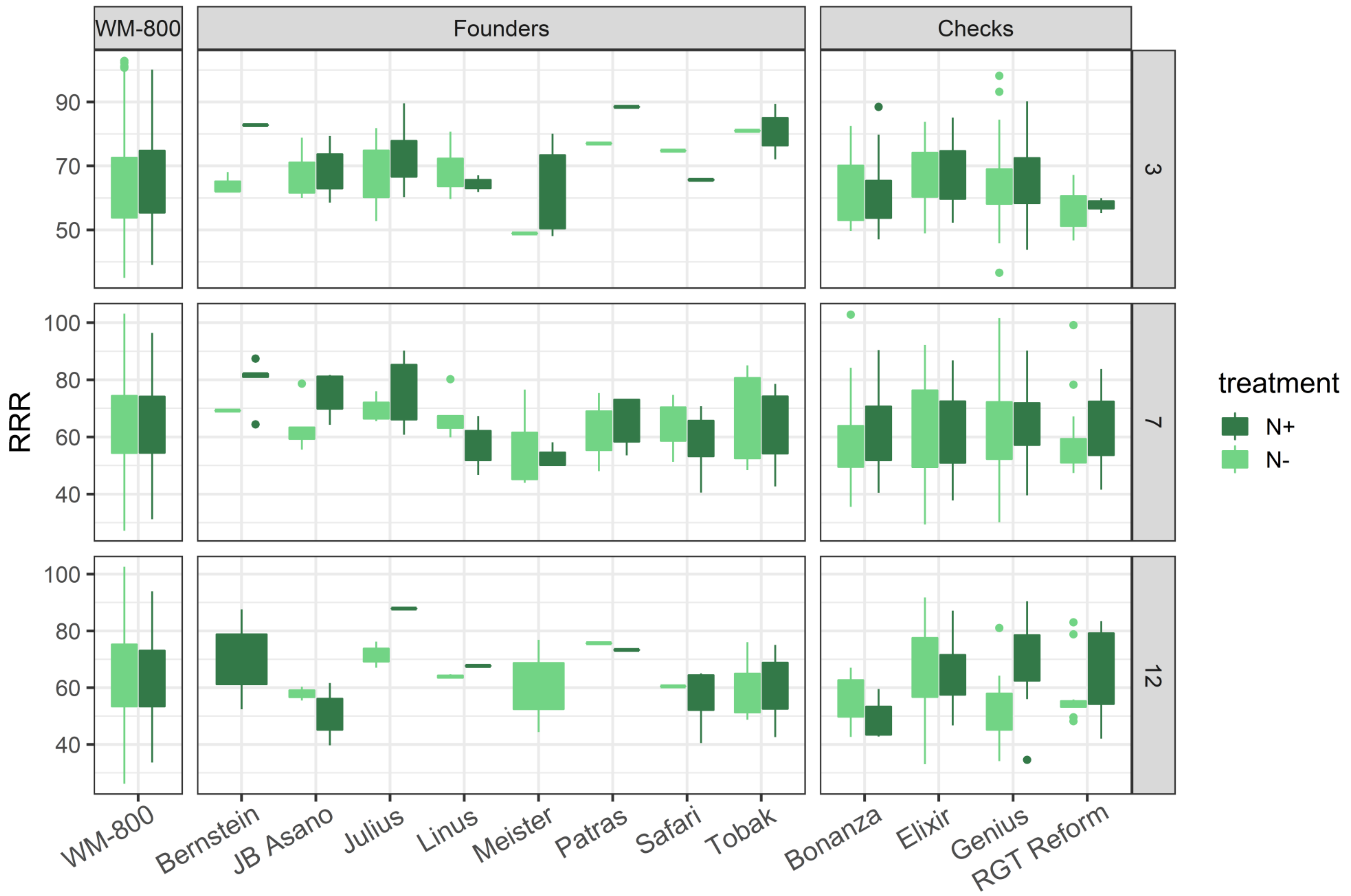

Figure S1 Box-Whisker-Plots comparing phenotypic varaition
between WM-800 lines, founders and check varieties

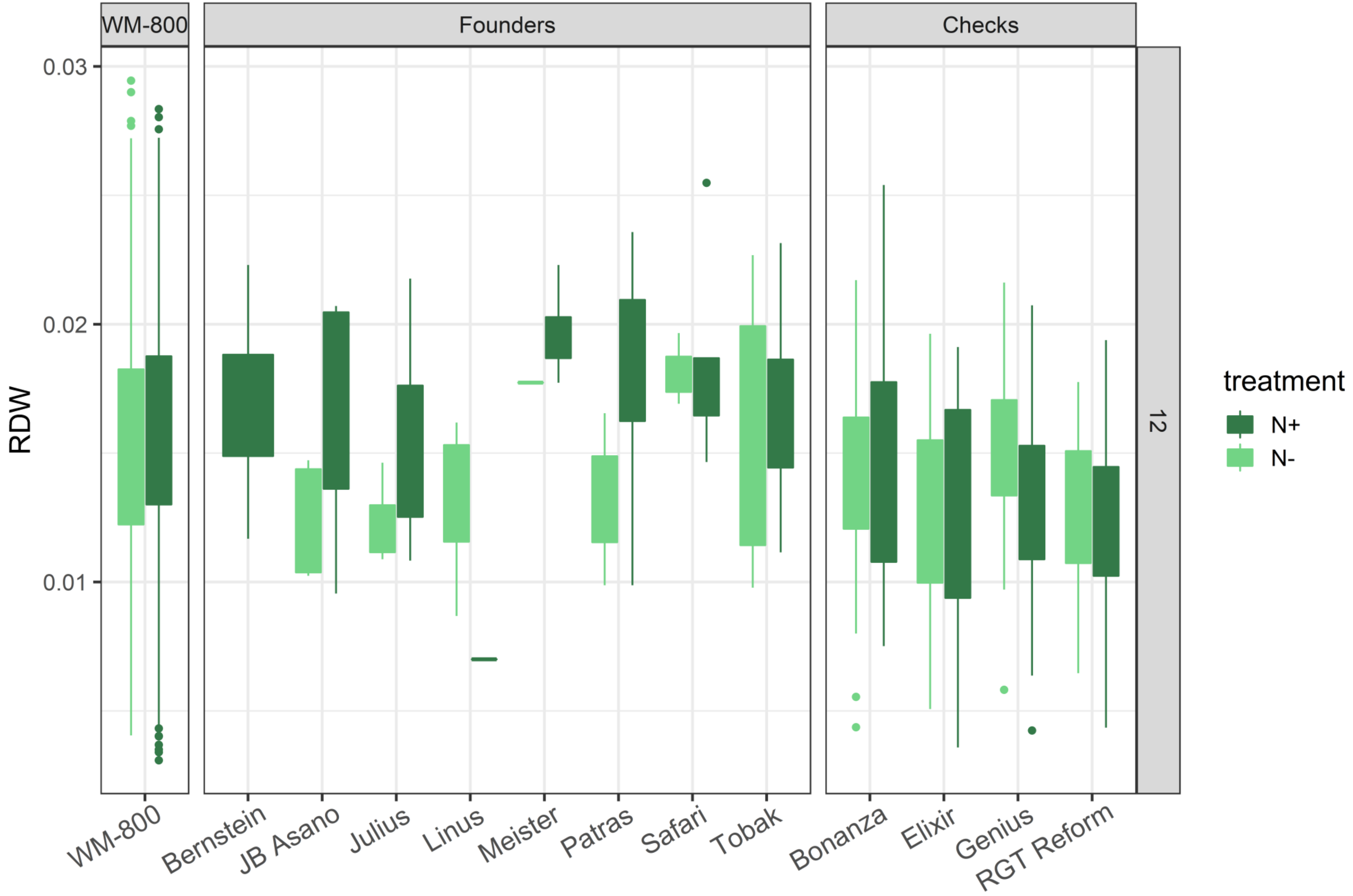

Figure S1 Box-Whisker-Plots comparing phenotypic varaition between WM-800 lines, founders and check varieties

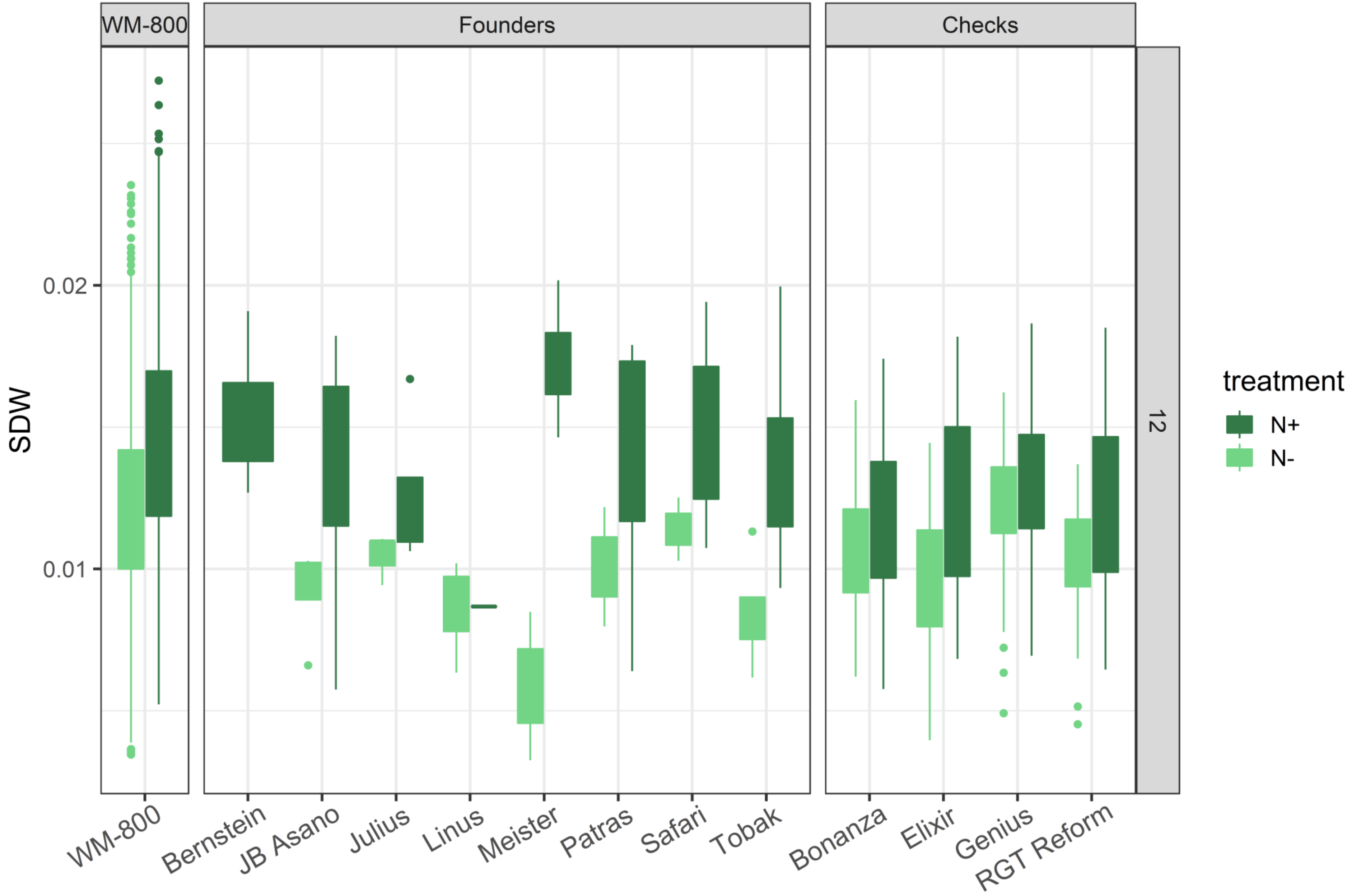

Figure S1 Box-Whisker-Plots comparing phenotypic varaition
between WM-800 lines, founders and check varieties

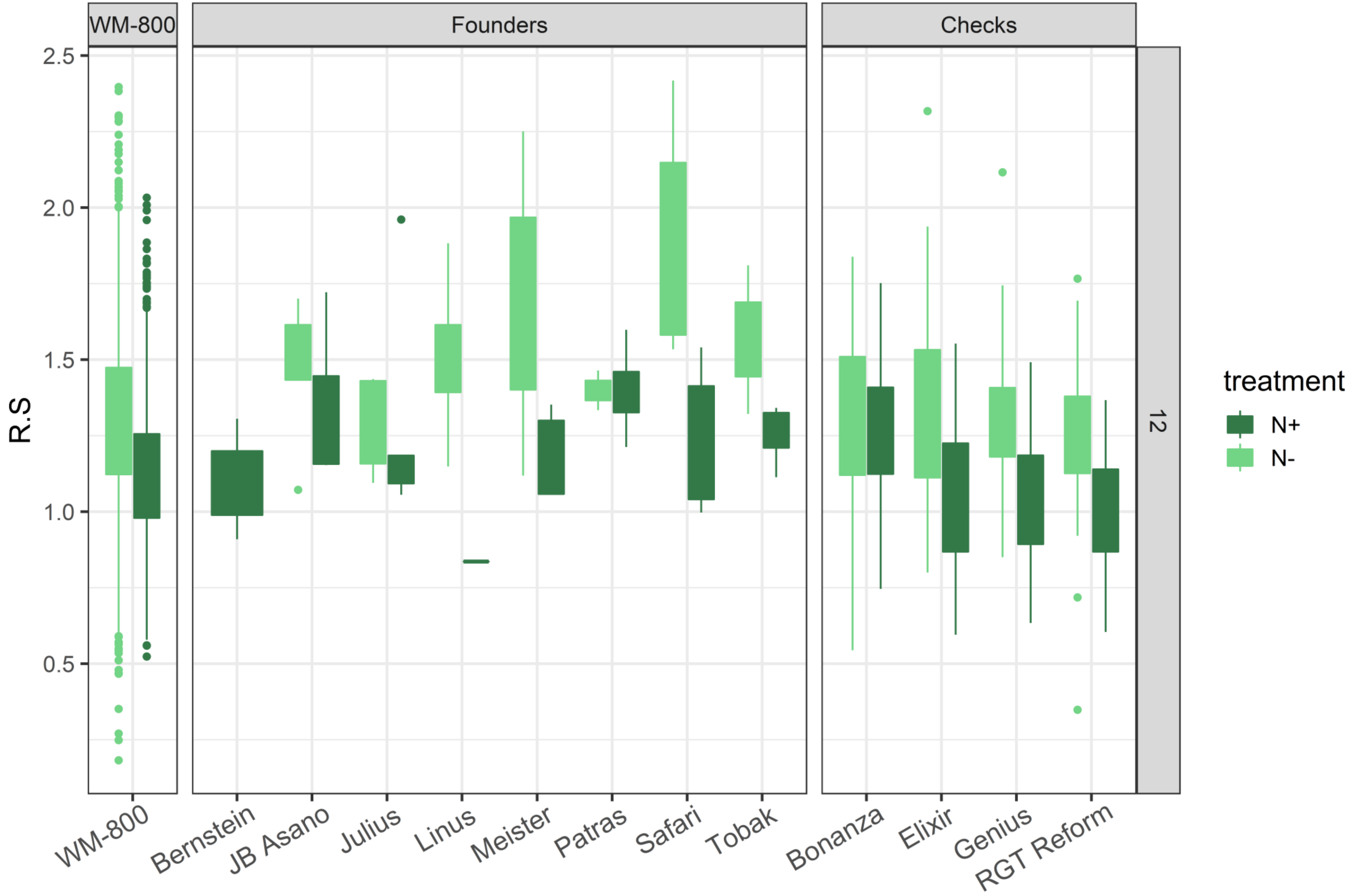

Figure S1 Box-Whisker-Plots comparing phenotypic varaition between WM-800 lines, founders and check varieties

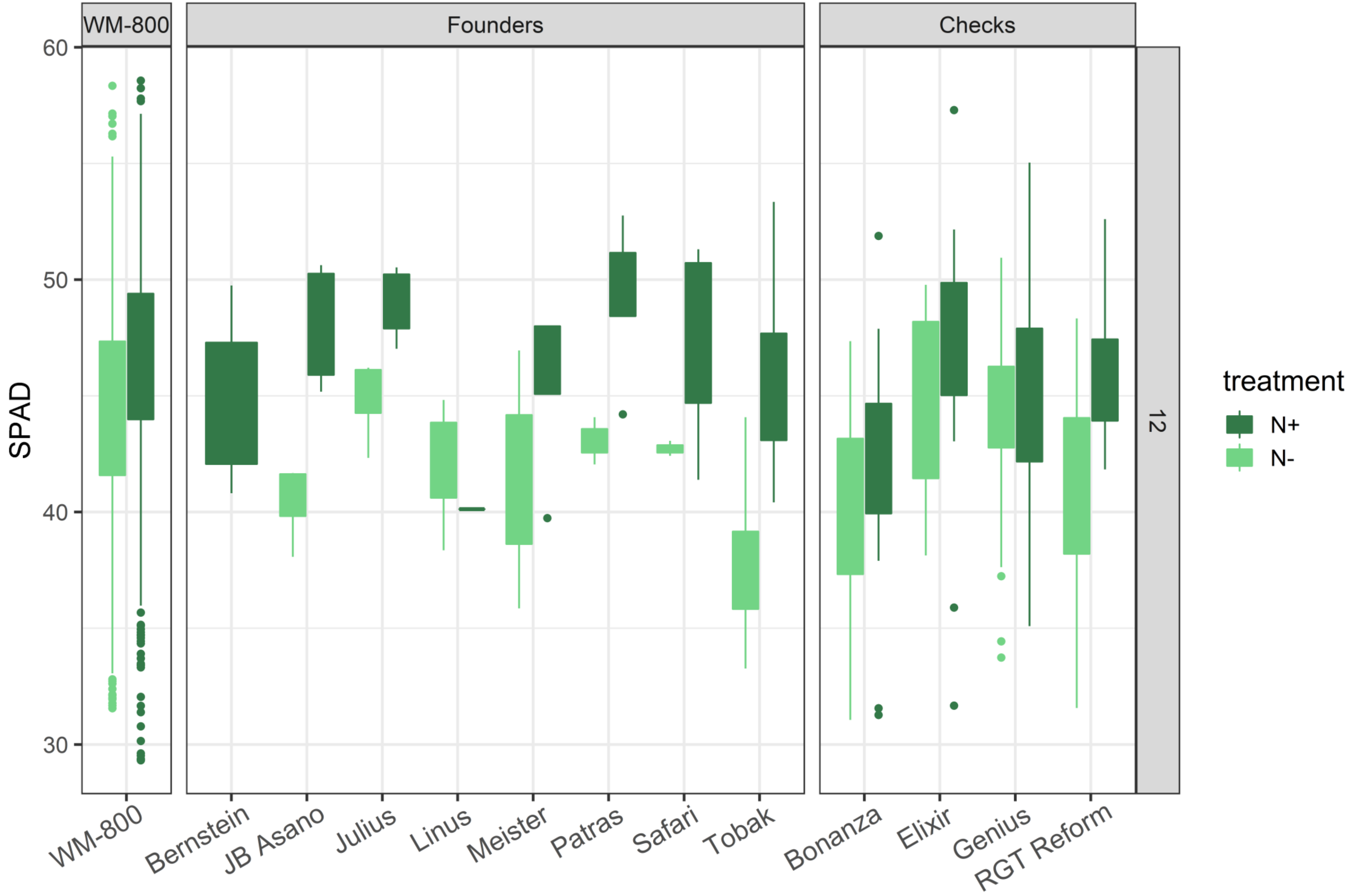

Figure S1 Box-Whisker-Plots comparing phenotypic varaition
between WM-800 lines, founders and check varieties

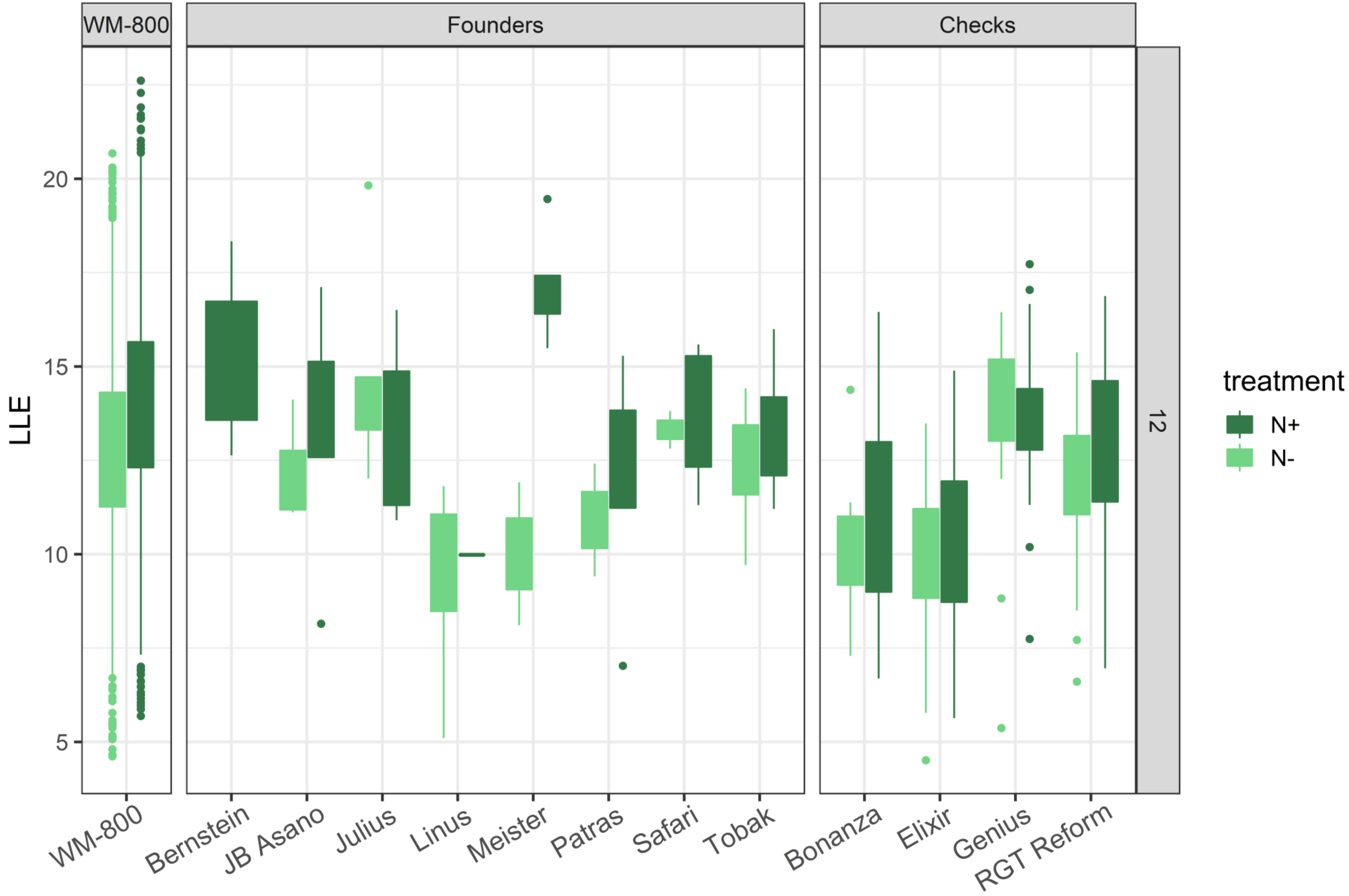

Figure S1 Box-Whisker-Plots comparing phenotypic varaition between WM-800 lines, founders and check varieties
