# Supplementary figures and images for: Unraveling Genomic Regions Controlling Root Traits as a Function of Nitrogen Availability in the MAGIC Wheat Population WM-800
Source: Plants (Basel). 2022 Dec 14;11(24):3520. doi: 10.3390/plants11243520 (PMC9785272; doi:10.3390/plants11243520)

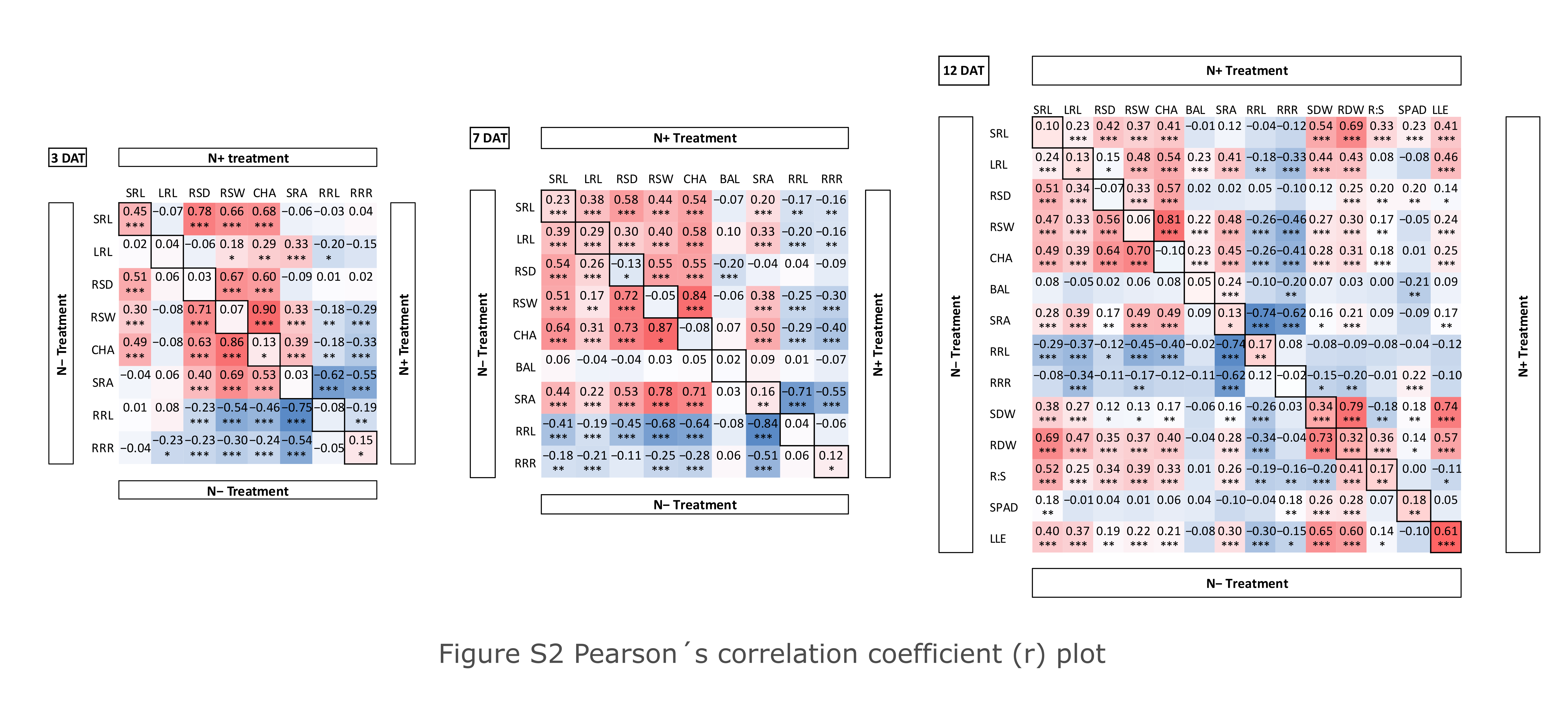

Supplement: Supplementary file 1 [file plants-11-03520-s001.zip › Figure S2 Pearsoní»s correlation coefficient (r) plot.png]
